# Crosstalk between intracellular and extracellular signals regulating interneuron production, migration and integration into the cortex

**DOI:** 10.3389/fncel.2015.00129

**Published:** 2015-04-14

**Authors:** Elise Peyre, Carla G. Silva, Laurent Nguyen

**Affiliations:** ^1^GIGA-Neurosciences, University of LiègeLiège, Belgium; ^2^Interdisciplinary Cluster for Applied Genoproteomics (GIGA-R), University of LiègeLiège, Belgium; ^3^Wallon Excellence in Lifesciences and Biotechnology, University of LiègeLiège, Belgium

**Keywords:** interneurons, cortex, migration, progenitors, nucleokinesis, branching

## Abstract

During embryogenesis, cortical interneurons are generated by ventral progenitors located in the ganglionic eminences of the telencephalon. They travel along multiple tangential paths to populate the cortical wall. As they reach this structure they undergo intracortical dispersion to settle in their final destination. At the cellular level, migrating interneurons are highly polarized cells that extend and retract processes using dynamic remodeling of microtubule and actin cytoskeleton. Different levels of molecular regulation contribute to interneuron migration. These include: (1) Extrinsic guidance cues distributed along migratory streams that are sensed and integrated by migrating interneurons; (2) Intrinsic genetic programs driven by specific transcription factors that grant specification and set the timing of migration for different subtypes of interneurons; (3) Adhesion molecules and cytoskeletal elements/regulators that transduce molecular signalings into coherent movement. These levels of molecular regulation must be properly integrated by interneurons to allow their migration in the cortex. The aim of this review is to summarize our current knowledge of the interplay between microenvironmental signals and cell autonomous programs that drive cortical interneuron porduction, tangential migration, and intergration in the developing cerebral cortex.

## Introduction

During mouse embryogenesis, cortical interneurons (cINs) are generated in the ventral subpallium. Distinct proliferative regions can be identified in this area, including the lateral ganglionic eminence (LGE), the medial ganglionic eminence (MGE), the caudal ganglionic eminence (CGE) and the preoptic area (POA). While the MGE, CGE and POA contribute to the generation of cortical interneurons (cINs) (Flames et al., 2007; Rubin et al., 2010), the LGE is mostly involved in striatal and olfactory bulb histogenesis (Yun et al., 2003). These distinct subpallial regions differ in progenitor domain composition and in the ability to generate IN subtypes characterized by specific networks of transcription factors. In addition to genetic programs, diffusing molecules also participate in shaping the timing, space and specificity of cIN subtype production. Indeed, at the earlier stages of corticogenesis, molecules acting as mitogens and morphogens induce genetic programs eventually leading to the expansion of proliferative regions, specification and maturation of cINs. In rodents, cINs migrate long distances to reach their final destination. Again, they are under control of genetic programs and signaling cascades triggered by extracellular cues that work together to produce a synchronized, harmonious and directed movement toward the cortex. At the cellular level, these informations are integrated and translated by the cytoskeleton into appropriate cellular behavior. After settling at their final location, cINs integrate and organize in coherent networks. Here we will review the current understanding of how genetic programs intermingle with extracellular signaling pathways to achieve the production, migration and network integration of INs in the cortex.

The exact number of cIN subtypes remains debated, mainly due to the diversity in their morphological, molecular and functional properties (Petilla Interneuron Nomenclature et al., 2008). In this review, a simplified nomenclature combining molecular and physiological properties of cINs will be used. According to this nomenclature (Gelman and Marin, 2010), the large variety of cIN subtypes will fall in one of four major groups: (a) fast spiking, parvalbumin (PV)-expressing cINs; (b) burst spiking or adapting non-fast spiking somatostatin (SST)-expressing cINs; (c) non-fast spiking and fast adapting calretinin (CR)- and/or vasointestinal peptide (VIP)-expressing cINs; (d) rapidly adapting neuropeptide Y (NPY)- and/or reelin-expressing cINs. Most studies discussed here used rodents as experimental model. They have been useful in the understanding of the physiopathology underlying cortical interneuron development. We will finalize by giving example of how these findings fit with what starts being known about the production, migration and cortical integration of GABAergic neurons in primate and human.

## Generation and specification of cortical interneurons

### Role of morphogens in establishing ventral identity

**Figure 1:**
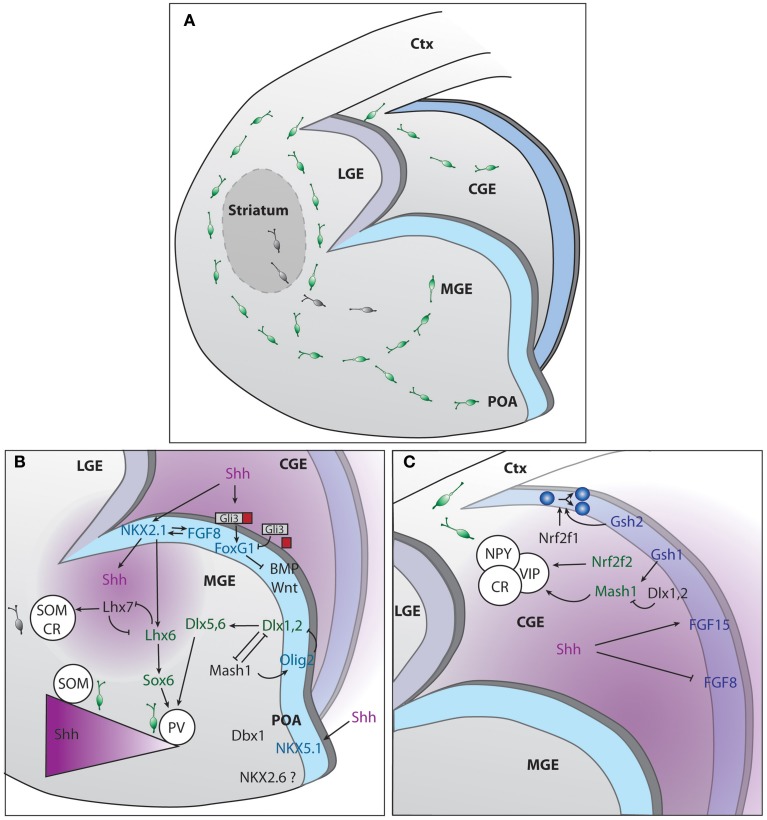
**Genetic regulation of cINs production in the different compartments of the subpallium. (A)** Representation of the developing mouse brain with the different subpallium structures and interneurons migration routes. **(B)** Signaling cascades regulating cIN production and specification, in the MGE and POA. **(C)** Signaling cascades regulating cIN production and specification, in the CGE. The two main sources of SHH, ventricles and mantle zone, are depicted in purple.

MGE histogenesis starts at around embryonic day (E) 9, followed by the generation of LGE at E10 and CGE at E11 (Smart, 1976). GEs histogenesis requires a complex interplay between morphogens and transcription factors to ventralize the structure and promote IN production. Sonic hedgehog (SHH) and fibroblast growth factors (FGFs) contribute to the dorso-ventral patterning and subpallium development (Jessell, 2000; Briscoe and Ericson, 2001; Ingham and McMahon, 2001). *Shh* is widely expressed in the prospective MGE by E9 and by E12 its expression spreads to the mantle zone of the MGE and POA (Echelard et al., 1993). Cell responsiveness to SHH greatly depends on the action of the transcription factors of the GLI family that can, in their constitutively cleaved forms, act as transcriptional repressors in the absence of SHH signaling. Conversely if uncleaved, they function as transcriptional activators in the presence of SHH (Bai et al., 2004; Pan et al., 2006). The current understanding postulates that SHH signaling is required to counteract GLI3 repressor activity therefore contributing to the positioning of the dorso-ventral boundary (Figure 1). At early stages of brain development, SHH prevents dorsalization of the ventral telencephalon, allowing the subsequent formation of the GEs. This is in contrast with the role of GLI proteins in the spinal cord where these factors act as activators by directly promoting ventral patterning (Rallu et al., 2002; Bai et al., 2004). Downstream effectors of SHH signaling are also required for ventral development. They include smoothened, the low-density lipoprotein receptor-related protein 2 (LRP2) or megalin and the multifunctional transmembrane protein Cdo (Fuccillo et al., 2004; Spoelgen et al., 2005; Zhang et al., 2006). In *Shh* knockout mouse models, rescue of the ventral telencephalon by compound *Gli3* removal gives the indication that other genes might act independently or downstream of *Shh* (Rallu et al., 2002). Several studies provide evidence that FGFs act downstream of SHH and can directly induce ventral gene expression in dorsal telencephalic explants when SHH signaling is inhibited (Aoto et al., 2002; Ohkubo et al., 2002; Kuschel et al., 2003; Gutin et al., 2006; Rash and Grove, 2007). Since SHH promotes *Fgf* expression (Martynoga et al., 2005), FGFs are considered mandatory effectors of SHH signaling (Figure 1). The forkhead G1 factor (FOXG1) is the main generator of direct ventralization within the forming ventral telencephalon as it induces expression of *Fgf8* (Martynoga et al., 2005) in the MGE or *FGF15* in the CGE (Borello et al., 2008). *Foxg1* acts in concert with FGF signaling, forming a positive feedback loop (Shimamura et al., 1995; Martynoga et al., 2005). *Foxg1* expression is independent of the direct action of SHH but impaired in the absence of *Shh* due to the increased repressor activity of GLI3 (Rash and Grove, 2007).

### Transcription factors acting in concert with morphogens in the MGE

The MGE contributes to the production of 50–60% of the total population of cINs in the mouse (Pleasure et al., 2000; Butt et al., 2005; Wonders and Anderson, 2006). On top of the hierarchical MGE SHH- and FGF-dependent organizers is *Nkx2-1* transcription factor (Sussel et al., 1999; Gutin et al., 2006; Storm et al., 2006; Fogarty et al., 2007; Xu et al., 2008, 2010). *Nkx2-1* itself maintains *Shh* expression within the early MGE, a process depending on FoxA2/HNF-3β transcription factor (Sussel et al., 1999) (Figure 1). Moreover, early removal of *Nkx2.1* from MGE progenitors re-specifies INs into early LGE medium spiny neuron identity, while its late removal leads to acquisition of CGE IN profile (Butt et al., 2008). *Nkx2*-1 is no longer detected in mice that lack expression of both *Fgfr1* and *Fgfr2* (Gutin et al., 2006). The analysis of the expression of several transcription factors within the ventricular zone (VZ) of the MGE have led to the proposal that this region can be compartmentalized into five different progenitor domains (Flames et al., 2007). The dorsal region of the MGE (dMGE) preferentially gives rise to somatostatin (SST)-expressing cINs. In contrast, the ventral part of the MGE (vMGE) was shown to mostly generate paravalbulin (PV)-expressing cINs. Since *Nkx2-1* expression promotes the specification of both SST and PV cIN subtypes, it was suggested that the SHH gradient determines the final fate of interneurons. High levels of SHH signaling would favor the generation of SST-expressing cINs at the expenses of PV-expressing cINs (Xu et al., 2010). Consistently, high expression levels of SHH effectors were found in the dMGE (Wonders et al., 2008). More recently, it was demonstrated that neurons from the mantle zone are an additional source of SHH (Flandin et al., 2011). They play a relevant role in maintaining a high SHH gradient in the dMGE, far away from the VZ. SHH production by mantle neurons was shown to require expression of *Lhx6* (Flandin et al., 2011), a direct target of *Nkx2-1*, also implicated in PV or SST fate acquisition. In the absence of *Lhx6*, NPY fate was promoted at the expenses of PV or SST (Liodis et al., 2007; Zhao et al., 2008). It was also shown that disruption of the gene encoding *FGF receptor 1 (Fgfr1)* lead to a loss of *Lhx6* and *Lhx8(7)* expression, both necessary for the formation of MGE-derived INs (Fragkouli et al., 2005; Gutin et al., 2006; Liodis et al., 2007) and maturation of PV-cINs (Smith et al., 2014). Other genes act downstream of or in concert with *Nkx2-1*. For example, high levels of *Lhx8(7)* expression shifts the fate of cINs toward globus pallidus GABAergic neurons and into cholinergic INs from the striatum (Zhao et al., 2003; Fragkouli et al., 2005). *Sox6* expression is required for the generation of the appropriate number of PV and SST INs as demonstrated in studies using *germline* and conditional knockout mice (Azim et al., 2009; Batista-Brito et al., 2009). In these mice models, a concomitant increase of NPY cINs was observed (Azim et al., 2009; Batista-Brito et al., 2009). *Sox6* acts downstream *Lhx6* and is expressed continuously within the MGE from postmitotic progenitor stage until adulthood and is implicated in the placement and maturation of PV and ST cINs (Batista-Brito et al., 2009). *Dlx* genes also contribute to cINs specification and maturation. *Dlx* genes expression is temporally regulated, following the sequence: *Dlx2, Dlx1, Dlx5, and Dlx6* (Liu et al., 1997; Eisenstat et al., 1999). *Dlx1/2* gene seems to be particularly important for the acquisition of SST, calretinin (CR), NPY and reelin fates (Cobos et al., 2005) as its absence leads to an abnormal expression of cortical markers in the ventral telencephalon (Long et al., 2009a,b). The expression of a wide range of transcription factors in the MGE progenitors from the VZ and subventricular zone (SVZ) as well as non-transcription factor proteins involved in migration and cortical integration are also under the control of *Dlx1/2* (Long et al., 2009a). For example, *Dlx1/2* has a repressor activity over *Arx* transcription factor, required for MGE differentiation. In their absence, cIN migration is blocked, resulting in accumulation of cells in the GEs and reduced numbers of INs in the cortex (Colombo et al., 2007). In addition, *Dlx1/2* genes are also required for the delayed expression of *Dlx5/6* genes (Anderson et al., 1997; Yun et al., 2002; Long et al., 2009b), particularly important for the establishment of PV subtype identity (Wang et al., 2010). Furthermore, *Dlx1/2* tightly control the generation of oligodendrocytes in the forebrain by repressing *Olig2* (Petryniak et al., 2007). More precisely, the choice between neuronal and glial fate involves cross-regulation between *Mash1* or *Acsl1* and *Dlx* genes. *Mash1* binds to and represses the regulatory DNA elements in the intergenic region of *Dlx1/2* (Parras et al., 2007). In *Mash1* mutants, *Dlx1/2* expression is expanded in the VZ/SVZ (Casarosa et al., 1999; Horton et al., 1999; Yun et al., 2002; Poitras et al., 2007). Conversely *Mash1* expression is increased in the VZ/SVZ of *Dlx1/2* mutants (Yun et al., 2002). Thus, the role of *Mash1* consists in restricting the number of *Dlx*-expressing progenitors (Petryniak et al., 2007).

### Transcription factors and morphogens shaping the generation of cINs in the CGE

CGE contributes to the generation of 30–40% of all cortical interneurons. Several studies have demonstrated that CGE derived interneurons acquire either a CR and/or VIP (Pleasure et al., 2000; Butt et al., 2005) or reelin identity (Miyoshi et al., 2010). *Gsh* or *Gsx* homeobox TFs act at the top of the genetic network involved in CGE cell specification (Figure 1). *Gsh2* is particularly relevant for the generation of CR bipolar cINs (Xu et al., 2010). Interestingly, *Gsh1* and *Gsh2* are co-expressed but have antagonist functions within the CGE, *Gsh2* promoting progenitor state and *Gsh1* promoting neuronal differentiation (Pei et al., 2011). Interestingly, the control of the choice between proliferation and differentiation by *Gsh* genes seems to involve the downstream target *Mash1* (Fode et al., 2000). In *Mash1* loss of function there is premature differentiation of progenitors located in the SVZ and precocious expression of *Dlx* genes (Casarosa et al., 1999; Yun et al., 2002), downstream effectors. On the other hand, overexpression of *Mash1* contributes to cell type specification (Fode et al., 2000). *Dlx1* and *Dlx2* are co-expressed in subsets of progenitor cells and contribute to cell maturation by downregulating *Gsh2/Mash1* (Yun et al., 2002). Other CGE transcription factors include *Nrf2f1and Nrf2f2 or Couptf1* and *Couptf2*, respectively, as well as *SP8*. These genes are however not exclusive from CGE, as they have been identified in the dMGE and POA (Lodato et al., 2011a). *Nrf2f1* is required for proper progenitor proliferation and necessary for generation of interneuron diversity in the cortex (Lodato et al., 2011a). *Nrf2f2* is important for directing interneurons through a caudal migratory path (Cai et al., 2013). *SP8* function in the hierarchy of CGE specification/maturation is yet unknown (Ma et al., 2012).

### The POA produces a reduced number of diverse cIN subtypes

The POA is the most ventral region of the developing subpallium and it has been shown to generate around 10% of GABAergic INs. Using a Cre line driven by *Nkx5-1* or *Hmx3*, a gene exclusive from POA, gives rise to a small population (around 4%) of multipolar GABAergic cells (Gelman et al., 2009). Another 5% of total INs is also produced by progenitors present in this region, characterized by their expression of the transcription factor Dbx1. Fate mapping and *in utero* transplantation demonstrated that POA generates diverse cINs subtypes (Gelman et al., 2011). In terms of molecular markers expression, the cells generated by the POA resemble the ones originating from the CGE (Gelman et al., 2009). *Shh* and *Nkx2-1* but not *Lhx6* are also expressed in the POA (Flames et al., 2007). *Dbx1* and *Nkx6-2* are respectively markers of the dorsal and ventral POA (see Figure 1). The function of these genes remains, however, elusive.

### Progenitors and proliferation in the ventral subpallium

Ventral telencephalon expansion and generation of a great diversity of cINs relies on the proliferation of pools of progenitors. The molecular rules governing cell proliferation in the ventral telencephalon as well as the characterization of the distinct cIN progenitor behavior has just started to be unveiled. For some time it was anticipated that GE progenitors would display a proliferative behavior similar to progenitors in the cerebral cortex (Ross, 2011). This view relied on anatomical and cumulative bromodeoxyuridine (BrdU) experiments. These studies were important as they served identifying both VZ and SVZ as two distinct proliferative compartments (Sheth and Bhide, 1997). The lack of selective markers for the SVZ and the superposition of proliferating and migrating cINs hampered for some time detailed studies aiming at characterizing the cellular biology of cell division of ventral progenitors. Improvement of molecular tools and imaging techniques overcame these limitations. For example, an elegant study by Brown and colleagues used a clonal approach to understand how cINs were generated within these regions. Low concentration of retrovirus expressing GFP was injected in the ventricle of E11 embryos. In order to specifically infect INs progenitor cells, the virus entry receptor was expressed under the control of *Nkx2*-1 (Brown et al., 2011). They found that cINs are produced as spatially organized clonal units and clonally related INs form spatially isolated cluster in the neocortex. They identified the presence of radial glia (RG) in the VZ of MGE and POA that undergo interkinetic nuclear migration and divide asymmetrically in the VZ to self-renew and produce intermediate progenitors (IPs) or differentiating cINs. Using time-lapse microscopy, Pilz et al. (2013) proposed a more complex hierarchical classification for ventral progenitors. RG cells sit at the base of this classification and divide asymmetrically to generate both an amplifying and a self-renewal branch. These cells give rise to short neural precursors (SNPs). Both RG and SNPs generate subapical progenitors (SAPs) which in turn divide to produce basal radial glia (bRG) or basal progenitors (BPs). Basal radial glia and BPs contribute to the great SVZ expansion. *Mash1* levels were shown to control the numbers of SAPs (Pilz et al., 2013). Although this study was performed in the LGE, such hierarchical complexity might be expected for the entirety of the ventral telencephalon.

### Molecular regulation of ventral proliferation

Studies performed by Vidaki et al. (2012) showed that classical proteins displaying a role in dorsal proliferation, such as Ras-related C3 botulinum toxin substrate 1 (Rac1), are also important regulators of *Nkx2-1*-expressing MGE progenitor division. In the absence of Rac1, cyclin D proteins levels are reduced and similarly low levels of Retinoblastoma (Rb) phosphorylation is detected. Cortical interneuron progenitors are thus blocked from completing cell cycle (halted in G1 phase) and accumulate in the GEs. Interestingly, the lack of *cyclin D2* in SVZ progenitors lead to the production of lower number of PV but not SST cIN subtypes (Glickstein et al., 2007a,b, 2009), suggesting that cell division and cell fate acquisition are linked events (Glickstein et al., 2007b; Ross, 2011). Rb family proteins and the closely related protein p107, play a role in cell proliferation by regulating the activity of E2F transcription factors, notably E2F4 a transcription repressor (Trimarchi and Lees, 2002). Deficiency of E2F4 expression impairs the self-renewal of neuronal precursor cells (Ruzhynsky et al., 2007) and results in loss of ventral telencephalic structures. The underlying mechanism involves a dramatic loss of *Shh*, *Nkx2-1*, and *Dlx2* expression.

Acting extracellularly, morphogens such as SHH and FGFs can also potentially act as mitogens (Hebert and Fishell, 2008). SHH-mediated proliferation is regulated in space and time (Blaess et al., 2006). If progenitors are exposed to SHH during the peak of neurogenesis, it will enhance proliferation, whereas exposure during the post-neurogenic period maintains cells in the undifferentiated state (Rowitch et al., 1999). Downstream effectors of SHH regulating the cell cycle are N-*myc* (Kenney et al., 2003), *cyclin D1* (Kenney and Rowitch, 2000), *E2f1* and *E2f2* (Oliver et al., 2003). The induction of N-*myc* occurs through GLI proteins and its stabilization depends on phosphatidylinositol-3-kinase (PI3-K) (Kenney et al., 2004; Sjostrom et al., 2005). FGFs control cell cycle length mainly during G1 phase. In a cell culture model, addition of FGF2 results in G1 phase shortening and an increase in the number of proliferative divisions by E14-E16 (Lukaszewicz et al., 2002). *In vivo*, *Fgf8* controls ventral telencephalon size mainly in rostral regions (Storm et al., 2006). This effect mainly relied on the control over progenitor survival (Storm et al., 2006). Deletion of *Fgf3* in addition to *Fgf8* further decreased the telencephalic size, indicating that both genes act in synergy (Theil et al., 2008). *Fgf15* controls progenitor differentiation at earlier developmental stages by promoting cell cycle shortening and exit, an effect opposite of what was observed at later stages (Borello et al., 2008). Neurotransmitter receptors are another class of diffusible molecules that control cell proliferation in the developing telencephalon (Cameron et al., 1998; Nguyen et al., 2001; Owens and Kriegstein, 2002a,b). cIN precursors and progenitors appear to be sources of gamma aminobutyric acid (GABA) (Bellion and Metin, 2005). Indeed, the extracellular GABA concentrations in the GEs may be as high as 0.5 μM (Cuzon et al., 2006). Proliferating cIN precursors also display detectable levels of GABA synthetizing enzymes and functional GABA_B_ receptors (Maric et al., 2001) as well as GABA and chloride transporters (Laurie et al., 1992; Ma and Barker, 1995). Glutamate is also present in the telencephalic germinal zones where it acts as mitogen. In the cortex, both GABA and glutamate where shown to decrease proliferation in the SVZ probably by reducing DNA synthesis as a consequence of membrane depolarization and Ca^2+^ increase (LoTurco et al., 1995; Haydar et al., 2000), in opposition to what was observed on VZ progenitors (Haydar et al., 2000). Glutamate actions were found to be diverse and depend on the glutamate receptor subtype involved in the signaling. For example, DNA synthesis inhibition occurs when α-amino-3-hydroxy-5-méthylisoazol-4-propionate (AMPA)/ kainate (KA) receptors are activated (LoTurco et al., 1995). N-methyl-D-aspartate (NMDA) receptor-dependent signaling instead promotes proliferation of striatal neural progenitors (Sadikot et al., 1998; Luk et al., 2003). The ERK (Extracellular Signal-Regulated Kinase)-PI3K pathway is triggered downstream NMDA receptor activation to control proliferation of striatal progenitors (Luk et al., 2003). Variation in glutamate concentration (Haydar et al., 2000) and interaction between glutamate receptor-mediated and growth factors-mediates signaling pathways (Dobbertin et al., 2000) might further contribute for a differential responsiveness of distinct progenitors. The action of morphogens and/or mitogens can be disrupted by many environmental agents and by epigenetic modifications during the period of corticogenesis. It is thus of utmost interest to fully characterize the signaling cascades triggering ventral proliferation.

## Migration of cortical interneurons

**Figure 2:**
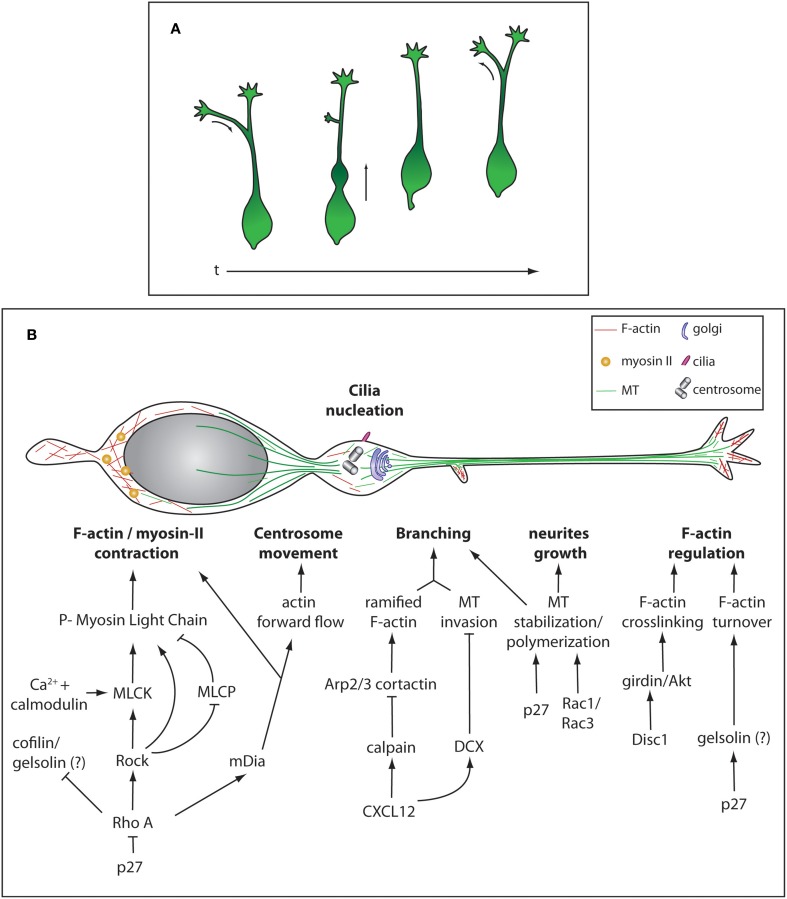
**Morphological remodeling during cIN migration and cytoskeleton regulation. (A)** Different stereotypical morphologies during of cIN migration over time. **(B)** Representation of the cytoskeleton and some cellular components of a migrating cIN. Cytoskeleton-regulating cascades are represented in specific cellular compartments, not meaning they are exclusive to these different compartments.

During development, cINs migrate over long distances to reach the cortex and settle within cortical layers. Migrating cINs are highly polarized cells harboring a branched and dynamic leading process that terminates in a growth cone-like structure. They also possess a membrane protrusion at the rear of the cell called trailing process. While migrating, cINs display a stereotyped cyclic movement (Figure 2A). First, there is an extension of branches emanating from the leading process and as one of the branches stabilizes, a transient swelling forms close to the cell body where the centrosome and Golgi apparatus are displaced (Bellion et al., 2005). Then, cINs move forward by sudden and fast nuclear translocation into the swelling, an event called nucleokinesis. The jumping behavior of cINs characterizes its migration pattern and distinguishes it from the treadmill-like movement observed in a large range of cells. Finally, the trailing process is retracted and the cycle repeats. cINs can significantly change the direction of migration by inverting polarity, the trailing process extending and becoming the new leading process while the older leading process undergoes retraction (Nadarajah et al., 2002) (Figure 2A). All these dynamic phases heavily rely on cytoskeleton remodeling.

### Molecular regulation of the cytoskeleton in migrating cINs

#### cINs migration and nucleokinesis

In cINs, a microtubule (MT) “cage” surrounds the nucleus and a large array of MTs connects this structure to the centrosome (Tanaka et al., 2004; Higginbotham and Gleeson, 2007; Godin et al., 2012) (Figure 2B). MTs are nucleated ahead of the nucleus to guide centrosome movement and docking to the cell membrane. At the membrane, the mother centriole is then able to grow a cilium allowing the cell to sense extracellular signals such as SHH (Baudoin et al., 2012). It was previously considered that the MT network was responsible for generating forces required for nucleokinesis, through dynein-dynactin directed motor movement. This type of MT-generated forces has been described for migration of projection neurons and cerebellar granule cells where MT-associated proteins Lissencephaly-1 (Lis-1) and Doublecortine (DCX) couple MT to the nucleus (Tanaka et al., 2004; Nasrallah et al., 2006; Tsai et al., 2007). Similarly, cINs depleted for Lis-1 show a reduced rate of migration (McManus et al., 2004). It was however demonstrated *in vitro* that the chemical destabilization of the MT network does not completely abolish nucleokinesis (Schaar and McConnell, 2005; Baudoin et al., 2008; Martini and Valdeolmillos, 2010). Instead, acto-myosin contractibility is necessary for nuclear movement, as shown by experiments in which non-muscle myosin-II-mediated contraction was blocked (Bellion et al., 2005; Schaar and McConnell, 2005; Baudoin et al., 2008; Martini and Valdeolmillos, 2010). Myosin-II is enriched behind the nucleus during nuclear translocation, where F-actin also shows a strong accumulation (Bellion et al., 2005; Schaar and McConnell, 2005; Martini and Valdeolmillos, 2010) (Figure 2B). Acto-myosin cytoskeleton is also necessary to promote centrosome separation from the nucleus upon swelling formation and for the dynamic remodeling of growth cones (Metin et al., 2006). Altogether, this strongly suggests that the forward movement occurring during nucleokinesis arises from pushing forces generated by acto-myosin contraction at the rear of the nucleus, together with limited MT- generated pulling forces (He et al., 2010; Steinecke et al., 2014a). Although well studied in the context of radial migration of projection neurons, the molecular cascade regulating acto-myosin cytoskeleton during tangential migration is not as well described. In many cell types the Rho family GTPases including Rho, Rac, and cell division control protein 42 homolog (CDC42) have been implicated in the regulation of acto-myosin contractility (reviewed in Heasman and Ridley, 2008; Govek et al., 2011). In cINs, modification of RhoA activation levels by loss of function of its inhibitor p27Kip1 leads to migration defects (Besson et al., 2004) due to myosin-II hyperactivation (Godin et al., 2012). Active RhoA, in its GTP bound state, regulates migration by activating the downstream kinase Rho-associated protein kinase (ROCK) and mDia but also by inhibiting cofilin, an actin severing enzyme (Kawauchi et al., 2006; Nguyen et al., 2006; Godin et al., 2012). ROCK promotes acto-myosin contraction using different mechanisms. It can directly phosphorylate Myosin Light Chain (MLC), inhibit Myosin Light Chain Phosphatase (MLCP) or activate Myosin Light Chain Kinase (MLCK) (Amano et al., 1996; Chrzanowska-Wodnicka and Burridge, 1996; Kimura et al., 1996; Ishizaki et al., 1997). MLCK activation leads to acto-myosin contraction by phosphorylating MLC. MLCK activity is modulated by its cofactor calmodulin, a calcium-activated protein (Gallagher et al., 1997). mDia is an actin nucleator that helps producing long filaments of actin fibers (Higashida et al., 2004) (Figure 2B). Interestingly, mutant mice for *mDia1* show impaired tangential migration, but no defects in radial cortical dispersion. In this animal model the anterograde actin flow that moves the centrosome forward as well as F-actin accumulation at the rear of the nucleus are impaired. In contrast, actin dynamics in the growth cone is normal (Shinohara et al., 2012). This argues for a differential regulation of cytoskeleton-generated forces in different subcellular compartments where ROCK and mDia cooperate to grant proper acto-myosin dynamics during cIN migration. F-actin turnover is a crucial parameter in acto-myosin dynamics as inhibition of F-actin severing was shown to stabilize the leading process and can lead to cell migration arrest (Chai et al., 2009a,b). In INs, partial inactivation of cofilin in mice lacking *p27* does not result in strong accumulation of F-actin, suggesting that F-actin severing can be mediated by redundant mechanisms, for example by the action of gelsolin (Godin et al., 2012) (Figure 2B).

#### cINs leading process, branching, and growth cone regulation

cINs navigate in their environment using a branched leading-process bearing dynamic growth cones. When a chemo-repulsive cue is sensed, the growth cone collapses and the branch is consequently retracted. In parallel, another branch is stabilized and it determines the new direction of migration (Martini et al., 2009). Leading process branching requires the coordination of both acto-myosin and MT networks. During this process a new membrane protrusion is formed thanks to the underlying ramified F-actin meshwork organized by cortactin and Actin-related proteins 2/3 (Arp2/3) (Spillane et al., 2011; Lysko et al., 2014). Then, invasion by unbundled and freely spreading MTs stabilizes the protrusion and allow the emergence of the branch. Recent evidence shows that fine-tuning of cIN branching is negatively regulated by CXCL12 or SDF-1 (Lysko et al., 2014). Binding of CXCL12 to its receptor CXCR4, results in decreased levels of cAMP and de-repression of calpain and DCX expression. This intracellular signaling has a double effect on the cytoskeleton: generation of straight F-actin fibers by calpain-mediated proteolysis of cortactin and MT bundling by DCX. Membrane protrusions are thus less likely to form in the absence of a branched actin meshwork and be stabilized by bundled MTs. Accordingly, hyper-branching is observed in cINs lacking *DCX* (Kappeler et al., 2006; Friocourt et al., 2007; Lysko et al., 2014) (Figure 2B). Proper regulation of MT dynamics is also essential for cIN tangential migration as neurite growth is depending on the establishment of new MT-networks. For example, in *p27kip1* mutant, neurite growth defects are observed and cannot be fully rescued by modulating the acto-myosin contraction mediated by the RhoA pathway (Godin et al., 2012). Interestingly it was shown that p27kip1 acts also as a MT-associated protein (MAP) thanks to its proline-rich domain that promotes MT polymerization both *in vivo* and *in vitro*. This indicates that MT polymerization is an essential parameter in modulating neurite growth and migration properties of cINs (Godin et al., 2012). Similarly, the establishment of a long leading process also requires MT stabilization. In *Rac1/Rac3* mutant mice, migrating cINs show a hyper-branched phenotype similar to the one observed in *Dcx* knockout mice as well as a reduced leading process length. This is also accompanied by MTs harboring less post-translational modifications (PTMs), frequent on stable and long-lived MTs. The phenotype was partially rescued by treating the cells with taxol, a MT stabilizing agent, indicating that establishment of a correct migration needs a minimal amount of stable MTs (Tivodar et al., 2014) (Figure 2B). Growth cone shape is modulated by both MT polymerization that generates pushing forces on the plasma membrane and by acto-myosin network underlying pulling forces on the leading edge. Actin undergoes polymerization and depolymerisation activity and acto-myosin contraction leads to the generation of an actin retrograde flow. The balance between these two networks will either allow growth or retraction of the leading process (Martini et al., 2009). Behind the leading edge, interactions between cell surface and migration substrate together with actin retrograde flow generate traction forces. Importantly, sectioning the leading tip or locally inhibiting acto-myosin contraction halts nucleokinesis, highlighting the role of this region in force generation (He et al., 2010). Actin cytoskeleton is regulated at the leading edge by Disc1 and cINs deficient for this protein accumulate less F-actin together with less phosphorylated Girders of Actin filaments (girdin) and Protein kinase B (Akt), crosslinkers of actin filaments (Steinecke et al., 2014a). Girdin targeting to to the leading tip requires interaction with Disc1 (Steinecke et al., 2014a). Mice mutant for *Disc1* or *Lis1*, display decreased level of acetylation, a marker of stable MTs in the growth cone. This suggests that stable bundles of MTs are not properly entering this structure and are important to grant the proper shape to the growth cone (Gopal et al., 2010; Steinecke et al., 2014a) (Figure 2B).

#### cINs migration substrate

Adhesion to a migration substrate allows cINs to generate acto-myosin forces as well as to organize cellular polarization and directionality. cINs seem to use cell-cell interaction to migrate along bundles of fibers of the corticofugal system invading the GEs (Metin and Godement, 1996). TAG-1 or contactin-2, a member of the immunoglobulin superfamily expressed by the corticofugal axons was proposed to mediate cell-cell contact between migrating cINs and corticofugal fibers as inhibition of TAG-1 leads to a strong reduction of cIN migration *in vitro* (Denaxa et al., 2001). However, *in vivo* TAG-1 does not play a role during migration cINs (Denaxa et al., 2005). In cINs, proteins displaying a role in adhesion such as talin, paxilin or Focal Adhesion Kinases (FAKs) are also calpain substrates (Franco and Huttenlocher, 2005). Calpain inhibition reduces migration speed despite the increased levels of F-actin (Lysko et al., 2014), implying that local adhesion turnover by cleavage is primordial for correct migration. Finally, N-cadherin, a homophilic cell adhesion molecule has been shown to be necessary during tangential migration (Luccardini et al., 2013). N-cadherin not only promotes cINs motility *in vitro* and *in vivo*, likely by promoting adhesion through the acto-myosin cytoskeleton (Giannone et al., 2009) but also contributes to the polarity maintenance. N-cadherin inhibition unable the centrosome/Golgi apparatus to enter the swelling and contributes to local defects of Myosin II contraction at the nuclear rear (Luccardini et al., 2013). Cell adhesion molecules play a relevant role for cINs migration as they are at the interface between the extracellular environment and the cytoskeleton.

### Migration of cINs: extracellular cues guide cINs movement

To allow proper directionality, integration of extracellular signaling is paramount. The extracellular cues are received by exploring the local environment thanks to stochastic branching of the leading process, and relevant cues are transduced at the level of cytoskeleton to grant the proper response of the cell: either extension of a new leading process is the right direction and/or retraction of existing branches (Britto et al., 2009). The first stage in which cINs are challenged by extracellular cues occurs within the GEs. During the differentiation process, newborn cINs need to be displaced from the VZ and SVZ and be driven toward the exit of the GEs. Two types of signaling drive the movement of cINs away from GEs. One has a chemo-repulsive effect to steer the cells in the good direction and the other stimulates cellular motility to enhance migration.

#### Chemo-repulsion within the GEs

Regarding the mechanisms mediating repulsion of INs from GEs, similarities were found with general mechanisms implicated in axonal guidance. Using a paradigm of *in vitro* brain slice preparation, Ephrin-A5/EphA4R interaction was shown to be necessary to control the repulsion response of cINs. The guidance molecule Ephrin-A5 is abundant in the VZ and cINs express the Ephrin receptor EphA4 (EphA4R) (Zimmer et al., 2008). In absence of Ephrin-A5, cINs were found ectopically invading the VZ, a phenotype rescued when the slices were treated with recombinant Ephrin-A5 (Zimmer et al., 2008). EphA4R-mediated forward signaling is also used by cINs to avoid migration toward the ventral region of the subpallium (Zimmer et al., 2011) as it also binds Ephrin-B3 present in the MGE and POA. It is noteworthy that EphA4R also promotes cIN migration trough EphrinA2 reverse signaling (Steinecke et al., 2014b). EphA signaling is integrated at the cellular level to remodel the actin cytoskeleton and steer cells in the right direction. Although little is known about the intracellular cascades downstream EphA4 in cINs, it generally acts in neurons through regulation of RhoA to stimulate growth cone collapse (Wahl et al., 2000). Moreover, in cINs, Src family kinases (SFKs) have been implicated in this process. Indeed, SFK inhibition results in the loss of Ephrin-A5/EphA4 repulsion (Zimmer et al., 2008). In mouse, four redundant SFKs have been identified (Thomas and Brugge, 1997) and they have been linked to the phosphorylation of numerous targets. They control the Rho family GTPase (Kullander and Klein, 2002) and inactivate cortactin (Huang et al., 1997; Weaver et al., 2001) resulting in decreased activity of Arp 2/3, actin filament breakdown and growth cone collapse (Weed and Parsons, 2001). Slit/Robo is another signaling pathway mediating chemo-repulsion in GEs and cINs express Roundabout homolog 1 (Robo1) (Bagri et al., 2002; Marillat et al., 2002), a receptor recognizing the ligands Slit homolog 1 and 2 (Slit1 and 2). These ligands and Robo1 are found in a complementary expression pattern in the VZ (Marin et al., 2002), and it was first thought that Robo/Slit signaling was at play to push cINs away from the VZ. However, mice deficient for *Slit1/2* do not show cIN migration defects (Marin et al., 2003). Instead, the lack of Slit ligands or removal of *Robo1* leads to aberrant striatal invasion by cINs (Andrews et al., 2006; Hernandez-Miranda et al., 2011). This indicates that on the way toward the GEs exit, cINs face a second set of signaling molecules that refine their migratory routes through the LGE, and around the forming striatum. It was previously shown that the striatum is a strong repulsive structure for cINs thanks to the expression of class 3 semaphorins: Sema3A and Sema3F (Marin et al., 2001). The effect of semaphorins is mediated in cINs by neuropilin (Nrps) and Plexin receptors. Sema3A transduces signal specifically trough Nrp1 and PlexinA1 receptors (McKinsey et al., 2013) and Robo1 modulate semaphorin-neuropilin/plexin expression levels (Hernandez-Miranda et al., 2011). It is noteworthy that Nrp1 is present exclusively in cINs so that they respond to the repulsive signals secreted by the striatum. *Nrp1* expression depends on the transcription factor Sip1 involved in *Nkx2-1* down-regulation and *Nkx2-1* is a *Nrp1* repressor (McKinsey et al., 2013). On the contrary, striatal INs generated by the same progenitors as cINs, do not express Nrp1 as they maintain highs levels of *Nkx2*.1 (Marin et al., 2000; Butt et al., 2008; Nobrega-Pereira et al., 2008). Since the signaling pathway downstream of Sema3A is highly conserved, although not directly studied in cINs, it is anticipated that similar mechanisms are at play in these cells. Sema3A signaling regulates actin-dependent growth cone collapse trough RhoA, ROCK and LIM Kinase (LIMK) activation to eventually phosphorylate cofilin and inhibits F-actin turnover (Aizawa et al., 2001; Causeret et al., 2004). RhoA activation also leads to increased actin contractibility (Zhang et al., 2003). In the axon growth cone, a crosstalk between actin and MT cytoskeletons has been observed as retrograde F-actin flow on filopodia can displace distal position of MTs (Schaefer et al., 2002). Sema3A signaling could also directly regulate MT dynamics by inducing double phosphorylation of Collapsin Response Mediator Protein 2 (CRMP2) by Glycogen Synthase Kinase 3B (GSK3B) and Cyclin-dependent Kinase 5 (CdK5). The double phosphorylated state of CRMP2 reduces its tubulin affinity and overexpression of the phospho- mutant CRMP2 decreases Sema3A-induced growth cone collapse (Uchida et al., 2005).

#### Migration of cINs: motogenic factors

Newly produced cINs are stimulated by diffusible molecules to enhance movement and migration. A wide range of factors, including neurotrophins and neurotransmitters (NTs), were found to increase cINs motility *in vitro* (Heng et al., 2007). Demonstration came from experiments in which recombinant Brain Derived Neurotrophic Factor (BDNF) or Neurotrophin-4 (NT4) where applied to organotypic slice cultures (Polleux et al., 2002). BDNF- or NT4 effects are mediated by the Tyrosin Kinase B Receptor (TrkBR) (Polleux et al., 2002). Downstream signaling likely involves PI3K and the modulation of actin cytoskeleton. However this effect was observed *in vitro* but not in mouse mutant for *TrkB* where number and position of cINs are unchanged (Jones et al., 1994; Polleux et al., 2002; Sanchez-Huertas and Rico, 2011). *In vivo*, neurotrophin-mediated signaling promoting cINs motility was also linked to the action of Glial Cell-Derived Neurotrophic Factor (GDNF), binding and activating GDNF Family Receptor α1 (GFRα1). GDNF-mediated effects did not result from expression of Rearranged During Transfection (RET) or Neural Cell Adhesion Molecule (NCAM), two classical co-receptor signaling molecules (Pozas and Ibanez, 2005; Canty et al., 2009). The downstream signaling involves a matrix-bound form of GDNF and syndecan-3. This interaction then activates SFK to promote neurite outgrowth. GDNF may thus promote cell migration by acting on the actin cytoskeleton via SFK and cortactin pathway (Yoneda and Couchman, 2003; Bespalov et al., 2011). Since *GFRα1* mutant mice shows perturbations in cINs regionalization and subtype differentiation, GDNF might have additional functions beyond modulating cINs motility (Pozas and Ibanez, 2005; Canty et al., 2009). The hepatocyte growth factor/scatter factor (HGF/SF) and its receptor MET were also reported to have a potent motogenic action on cINs *in vitro*. Mutant mouse for the *urokinase-type plasminogen activator receptor* (*uPAR*) that cleaves and releases the active form of HGF/SF (Powell et al., 2001) or mutant mice for *MET* (Eagleson et al., 2011) shows a decreased number of cINs in the cortex. The cell-autonomous effect of HGF/SF-mediated signaling was later questioned since MET is not found to be expressed in cINs *in vivo* but is rather found in projection neurons and their axonal fibers (Eagleson et al., 2011). Several NTs/neuromodulators have also been implicated in cINs migration. Ambient GABA is found in high concentration in the MGE and in the cortical migration streams (Cuzon et al., 2006). cINs express GABA_A_ and GABA_B_ receptors and as a result of an inverted chloride gradient, they respond to GABA by membrane depolarization (Owens et al., 1999) that triggers opening of L-type voltage-sensitive Ca^2+^ channels and induces Ca^2+^ transients (Bortone and Polleux, 2009). Antagonizing GABA_A_ receptor function prevents cINs from crossing the cortico-striatal barrier, leading to their accumulation at the pallial/subpallial boundary. Conversely, in experiments where exogenous GABA or diazepam are added to brain organotypic slices, a higher number of cINs were found exiting the GEs (Cuzon et al., 2006). Similarly, Glycine α2 receptor is also regulating cIN migration and in particular nucleokinesis by fine tuning acto-myosin II contraction (Avila et al., 2013). Activation of GlyRs by glycine leads to Ca^2+^ transients due to opening of voltage-gated Ca^2+^ channels and GlyRs α2 loss of function impairs cINs migration in the cortex. Glutamate signaling through AMPARs, located on the plasma membrane of migrating cINs also induces membrane depolarization and sodium influx (Metin et al., 2000; Manent et al., 2006). Blockade of AMPARs decreases cortical and hippocampal invasion by INs (Manent et al., 2006; Bortone and Polleux, 2009). Membrane depolarization and Ca^2+^ transients are thought to stimulate cINs motility as calmodulin bound to Ca^2+^ ions can interact and activate MLCK. In turn, MLCK phosphorylates Myosin Light chain II on serine 19 and promotes acto-myosin contraction (Metin et al., 2000; Bortone and Polleux, 2009). Another example is the motogenic effect mediated by dopamine. Ambient dopamine is secreted in the GEs and close to the lateral VZ by projecting thalamo-striatal axons in the neo-striatum. Dopamine receptors *D_1_* and *D_2_* are expressed by cINs and have opposite effects on migration (Ohtani et al., 2003). When selectively blocked, D_1_R induces activation of D_2_R and impedes migration. This indicates that D_1_R has migration promoting action and conversely D_2_R is rather a migration stop signal. Other ubiquitous signaling molecules such as adenosine have also been implicated in the modulation of cINs migration. However the underlying mechanisms are still unknown (Silva et al., 2013).

### Migration of cINs: chemo-attraction toward the cortex

As cortical INs are steered away from the VZ/SVZ and around the striatum, they are simultaneously attracted toward the cortex by other molecules to cross the cortico-striatal junction and invade the pallium. Neuregulin-1 (Nrg1) is a strong attractant for cINs and can have both short and long-range effects depending on the isoform recruited. CRD-Nrg1, is membrane bound and acts on neighboring cells. It is mainly expressed by LGE cells and contributes to the formation of a permissive migration corridor for cINs. It is sensed by cINs as they probe their environment and inhibition of their branching behavior leads to a decreased ability to follow Nrg-1 gradient (Martini et al., 2009). The downstream signaling involves recruitment and activation of associated tyrosine kinases related to the epidermal growth factor (EGF) receptor ErbB4. Lg-Nrg1 is a diffusible form expressed by the cortex that can act as a long-range attractant to guide cells toward the cortical regions as they exit the LGE. *In vivo*, the loss of either *Nrg-1* or *ErbB4* causes migration defects and reduces the number of INs in the cortex (Yau et al., 2003; Flames et al., 2004; Neddens and Buonanno, 2010). At the cellular level, the presence of a Nrg-1 gradient does not prompt cINs to reorient an existing branch but rather to sprout a new leading process in the direction and at an angle corresponding to highest concentration of the attractant. The signaling events downstream ErbB4 have not been completely characterized in cINs, but they seem to be ROCK-independent (Martini et al., 2009). Thanks to its tyrosine kinase activity, ErbB4 can activate several distinct signaling pathways including the ras/raf/MAPK or the PI3 kinase pathway (Scaltriti and Baselga, 2006). In ErbB4 can be cleaved in its intracellular domain and be targeted to the nucleus (Ni et al., 2001). Interestingly, ErbBs can also phosphorylate β-catenin and modulate Cadherin signaling pathways involved in actin cytoskeleton remodeling (Hazan and Norton, 1998; Behrens, 1999; Al Moustafa et al., 2002). Moreover, other signaling molecules participate to the attraction of cINs toward the cortex. For example, CXCL12 is secreted by proliferating cortical cells from SVZ and IZ and is an attractant molecule for migrating cINs (Tiveron et al., 2006; Stumm et al., 2007).

### Migration of cINs: the choice of the migratory route

Cortical invasion does not occur in a stochastic manner as cINs integrate and move in migratory streams. At earlier stages of cortical development, two streams can be identified in the pallium: one called intermediate zone (IZ) stream and located above the VZ/SVZ surface (Nadarajah et al., 2002) and the other positioned at the level of the marginal zone (MZ) and called MZ stream (Lavdas et al., 1999). Between E15 and E16, a third subplate (SP) stream forms between the IZ and MZ streams. Other routes have also been identified, for example a caudally directed stream arising from the CGE (Yozu et al., 2005). Although not depending on their birth place (MGE, CGE or POA), the choice of the migratory route by cINs does not seem to be random (Miyoshi and Fishell, 2011). Evidence comes from the observation that populations of cINs migrating along the different cortical streams do not show the same gene expression profile as revealed by microarray analysis on micro-dissected Gad67+ positive cells isolated from either IZ or MZ streams (Antypa et al., 2011). Some population-specific genes, such as Cdh8, Plxnd1, Sema5a, Robo 1 and 2 or the reelin receptor Dab1, play key roles in neuronal migration.

Moreover *in vivo* experimental approaches have also shed light on intrinsic cellular component that are implicated in cINs sorting between the different migration routes. For example, it was found that mutations in the *Rb* gene unable cINs to enter the MZ stream and redirects them to the IZ stream (Ferguson et al., 2005). This effect is cell autonomous as suggested by cINs transplantation experiments made on wild-type organotypic brain slices. A similar phenotype was found to be linked to glycine receptor α2 subunit (Glyα2R), present on the plasma membrane of migrating cINs. Activation of Glyα2R opens voltage-gated Ca^2+^ channel (Avila et al., 2013). Extracellular cues seem to indeed play a relevant role in sorting cINs trough the different migration stream. Mutation of both Netrin1 and α3β 1 integrin, in the cells serving as migration substrate for cINs, gives rise to major migration defects in the MZ stream (Stanco et al., 2009). Finally GABA signaling, in addition to its migration promoting role, seems to be also implicated in the choice of the migratory route during cortical migration. Upon selective blockade of metabotropic GABA_B_R, cINs where found to be displaced from the MZ and CP stream toward the VZ/SVZ compartments (Lopez-Bendito et al., 2003).

### Migration of cINs: molecular regulation controlling the timing of cortical invasion

In the streams, tangentially migrating cINs do not invade the cortical plate (CP) and this process is coordinated with projection neurons migrating radially to form the cortical layers. Avoidance of the CP do not require repulsive cues coming from the projection neurons but rather the formation of a permissive corridor in the MZ and IZ thanks to chemokine signaling. CXCL12 is secreted by proliferating cortical cells from SVZ and IZ and it is a strong attractant molecule for migrating cINs (Daniel et al., 2005; Tiveron et al., 2006; Lopez-Bendito et al., 2008). Two receptors for this chemokine were identified in interneurons, CXCR4 and CXCR7 (Tiveron et al., 2006; Lopez-Bendito et al., 2008; Wang et al., 2011) and they signal through different downstream cascades. CXCR4 signals through Gα(i/o) while CXCR7 transduces independently on heterodymeric G proteins (Wang et al., 2011). In immature MGE neurons, CXCR7 acts as potent activator of MAP kinase signaling required for ERK1/2 phosphorylation (Wang et al., 2011). *Cxcr7* expression in the cortical plate expression follows a dorsoventral gradient, opposite to *Cxcl12* gradient in the SVZ. It was proposed that CXCR7 may lower the concentration of CXCL12 in the CP, generating a gradient from MZ and SVZ to the CP. A gradient of CXCL12 would be important for the regulation of cortical invasion (Wang et al., 2011). Indeed, disruption of CXCR4 or CXCR7 function results in premature exit of cINs from their migratory streams and perturbs their laminar and regional distribution within the neocortex (Li et al., 2008; Lopez-Bendito et al., 2008; Tanaka et al., 2010).

## Integration OF cINs into cortical assemblies

Cortical neurogenesis generates a six-layered cortex and cortical neurons distribute within these layers in an age-dependent manner, deep layers being generated before upper layers. Neurons sharing the same layers exhibit similar patterns of connectivity (Dantzker and Callaway, 2000). The cortex is also organized vertically and cells sharing the same cortical column will be linked by extrinsic connectivity (Mountcastle, 1997). Interneurons have to integrate into cortical circuits in order to fit in these two patterns of organization. Two studies have shown that clonally related cINs are preferentially consigned to specific cortical layers or columns (Brown et al., 2011; Ciceri et al., 2013). cINs generated by clonally-related progenitors are however diverse as they express markers of distinct subtypes. Furthermore, cINs from the same cardinal class are able to form connections with a variety of synaptic partners (Kepecs and Fishell, 2014). From their birthdates, the first interneurons populating the cortex are generated in the MGE and express PV, SST or CR/SST (Butt et al., 2005; Wonders and Anderson, 2006). The second wave of cortical invasion generated by the CGE produces INs expressing VIP, CR/VIP, calbindin (CB) or choline acetyltransferase (ChAT) and NPY, (Yozu et al., 2004; Miyoshi et al., 2010). While pioneer cINs populate the cortex in an inside-out mode following the pattern of cortical integration of projection neurons (Miller, 1985; Fairen et al., 1986; Valcanis and Tan, 2003; Rymar and Sadikot, 2007), late-migrating cINs instead concentrate in supragranular layers, independently of their birthdate (Xu et al., 2004; Rymar and Sadikot, 2007; Miyoshi et al., 2010). Since the birthdate and birthplace are not general predictors of the final specification and lamination of cINs, the mechanisms determining the proper and site-specific integration of INs in the developing cortex need to be clarified. This raises the question whether intrinsic and/or extrinsic factors determine the cortical integration and lamination.

### Intrinsic factors regulating cINs integration into cortical layers

There is evidence that a maturational program exists and the behavior of cINs, at a given developmental stage, depends on their cellular age. In the cortical streams, cINs generated at different developmental stages co-exist and they exit the migratory streams at different time points even if the signaling regulating the exit from the streams is the same. Thus, cINs born earlier invade the cortical place before late-born interneurons (Lopez-Bendito et al., 2008). Further evidence supports this concept of intrinsic regulation. For example, it was found that the motility of interneurons in cortical slices gradually decreases as development proceeds and is almost abolished by the end of the first postnatal week (Inamura et al., 2012). Accordingly, late-born cINs transplanted in younger embryos settle in deep layers instead of occupying the expected superficial layers (Pla et al., 2006). The mechanisms explaining intrinsic regulation of neuronal migration remain elusive. It was suggested that the frequency of Ca^2+^ transients is reduced as the neuron completes its migratory program (Kumada and Komuro, 2004). Other studies proposed that the intrinsic regulation of motility might be linked to the expression of the potassium-chloride transporter KCC2. KCC2 could modulate cINs motility by reverting the chloride potential and thus reducing membrane depolarization upon GABA_A_ receptor activation to serve as a stop signal for migration (Bortone and Polleux, 2009; Inamura et al., 2012). This is in agreement with the observation that cINs up-regulate KCC2 chloride transporter as soon as they exit the tangential mode of migration and start their radial sorting in the cortex (Miyoshi and Fishell, 2011). Cell autonomous regulation also contributes to the survival of interneurons as they invade the cortical plate. Transplantation experiments revealed that many cINs undergo programmed cell death *in vivo* between postnatal day (P) 7 and P11. When transplanted in older cortices, younger cINs die by apoptosis later than resident cINs (Southwell et al., 2012).

### Extrinsic factors regulating cINs integration into cortical layers

Regional distribution of cINs seems also depending on extrinsic signaling. Elegant studies suggested, in the last past years, that molecular cues released by projection neurons contribute to the establishment of the laminar distribution of cINs (Hevner et al., 2004; Pla et al., 2006; Yabut et al., 2007; Lodato et al., 2011b). The works from Hevner (Hevner et al., 2004), Pla (Pla et al., 2006), and Yabut (Yabut et al., 2007) using the *reeler* mouse model showed that cINs distribution within the cortex mostly results from the aberrant organization of cortical layers rather than the loss of reelin signaling transduction in cINs. The subsequent work from Lodato et al. (2011b) further support this hypothesis. *Fezf2* mutant mice lack sub-cerebral projection neurons, while all other projection neurons are normally produced. They show that *Fezf2* depletion does not impair cINs specification but rather the lack of subcerebral projection neurons non-autonomously impairs the proper distribution of SST, PV but not CR cINs subtype. Delayed overexpression of *Fezf2* in *Fezf2* null mice leads to the production of ectopically located clusters of subcerebral projection neurons. Many cINs invade these aggregates while the number of INs recruited depends on the size of the ectopic aggregates. Local excitatory and inhibitory signals may also influence the final positioning of INs (De Marco Garcia et al., 2011; Lyons et al., 2012; McKinsey et al., 2013). For example, it was shown that attenuating the activity of specific cIN populations affects the migration and morphologic development of cIN (De Marco Garcia et al., 2011). A number of activity-dependent genes specifically expressed by cINs have been identified. These include *Dlx1, Elmo1, and Mef2c*. Moreover the observation that voltage-gated Ca^2+^ influx may induce *de novo* gene expression opens the possibility that local activity might regulate direct region-specific differentiation and maturation of INs (De Marco Garcia et al., 2011; West and Greenberg, 2011). Additional evidence came from studies showing that MGE-derived interneurons are able to integrate in pathological neural circuits (Martinez-Cerdeno et al., 2010; Braz et al., 2012).

## Conclusion

The recognition that many neurologic disorders such as schizophrenia, epilepsy and autism have components related to cIN development greatly prompted advances in this field. Postmortem analysis of the human brain and studies performed in primates strongly support the idea that human and primate cINs are produced in both dorsal and ventral regions (Fertuzinhos et al., 2009; Hansen et al., 2013; Ma et al., 2013). These studies have been instrumental to highlight the differences and similarities between in cINs generation across species, acknowledging more similarities than initially expected. These findings have given more credit to studies performed in rodents, designed to understand the genetic and molecular regulation underlying human pathology. For example, in the case of schizophrenia, *NRG1* and *ERB4* as well as DISC1 have been identified as susceptibility genes in human (Millar et al., 2000a,b; Corvin et al., 2004; Marin, 2012). Studies performed in rodents established a coherent outline of the biological causes of this human disease (Flames et al., 2004; Fazzari et al., 2010). How developmental perturbations of cINs lead to a specific brain disorder remains unclear although it has been proposed to depend, to some extent, on the cIN subtype affected (Marin, 2012). Thus, understanding the role of different classes of cINs and the neuro-circuitry they modulate will be relevant for unraveling the genetic causes of human diseases and to propose effective therapeutic approaches.

In the last decades, important advancements were made in the understanding of how INs are generated and function into networks. However, the fine regulation of cIN development might not be explained merely by genetic programs and extracellular signaling. Indeed, a fully new dimension of regulation, including post-transcriptional and post-translational modifications (PTMs) might be at play during corticogenesis. Post-transcriptional and PTMs can oppose or reinforce genetically-encoded and/or extrinsically-mediated signaling. A recent study explored the role of miRNA in distinct aspects of cINs development (Tuncdemir et al., 2014). In this study, *Dicer*, an enzyme required for miRNA processing and maturation was genetically deleted from MGE-derived cINs. Interestingly, the loss of miRNAs had no effect on cell proliferation and initiation of tangential migration but affected the transition from tangential to radial migration as well as modified the survival and maturation of cINs. Upon *Dicer* knockdown there was a precocious expression of cINs markers such as *SST, GAD65 and NPY* and at a later stage. MGE-derived cells failed to express markers of their subtype identity. PV-expressing cINs seem particularly affected by the absence of miRNAs. Furthermore, a different profile of gene expression related to differentiation, cellular interaction and survival was found in *Dicer* knockout mice. The role of miRNA signaling was also recently tested in a mouse model of schizophrenia and bipolar disorder (Toritsuka et al., 2013). This study showed a direct link between the 22q11 micro deletion and defects in cortical and hippocampal interneuron migration, relying on functional abnormalities in CXCR4/CXCL12 signaling. Mechanistically, Toritsuka et al. (2013) demonstrate the pivotal role of *DiGeorge syndrome critical region gene 8* (*Dgcr8*)-mediated in miR-200a regulation necessary for the maintenance of CXCR4 levels. PTMs occur in most proteins and often contribute to their functions and subcellular behaviors. Not many studies were developed to investigate the contribution of PTMs on brain development but there is evidence that they can regulate different aspects of cIN development. For example, it was demonstrated that acylation of SHH N-terminus changes its efficacy as a signaling molecule and greatly enhances its ability to ventralize early LGE progenitors (Kohtz et al., 2001). A C-terminal cholesterol modification has also been identified on SHH relevant for SHH tethering to the cell surface (Porter et al., 1996). At the functional level, these PTMs were showed to be primordial in determining SHH “short range” or “long range” function (Burke et al., 1999). Another example is the polysialylation of the neural cell adhesion molecule (PSA-NCAM), important to control the timing of the perisomatic GABAergic synapse maturation in the mouse cortex (Di Cristo et al., 2007). Di Cristo and colleagues showed that premature removal of PSA in the visual cortex resulted in precocious maturation of perisomatic innervation by PV basket cINs. Interestingly, polysialytransferases have been implicated in schizophrenia (Arai et al., 2006; Tao et al., 2007; Isomura et al., 2011). In light of this, it seems highly pertinent to pursue on understanding how genes, signaling molecules and environment communicate to shape brain development and function.

### Conflict of interest statement

The authors declare that the research was conducted in the absence of any commercial or financial relationships that could be construed as a potential conflict of interest.

## Abbreviations

CGE: caudal ganglionic eminence
cIN: cortical interneuron
CP: cortical plate
CR: calretinin
dMGE: dorsal medial ganglionic eminence
F-actine: fibrilar actine
G-actine: globular actine
IP: intermediate progenitor
IZ: intermediate zone
LGE: lateral ganglionic eminence
MGE: medial ganglionic eminence
MT: microtubule
MZ: marginal zone
NPY: neuropeptide Y
POA: preoptic areo
PTM: post-translational modification
PV: parvalbumin
SAP: subapical progenitor
SNP: short neuroal precursor
SP: subplate
SST: somatostatin
SVZ: subventricular zone
VIP: vasointestinal peptide
vMGE: ventral medial ganglionic eminence
VZ: ventricular zone.

## References

[B1] Aizawa H. Wakatsuki S. Ishii A. Moriyama K. Sasaki Y. Ohashi K.. (2001). Phosphorylation of cofilin by LIM-kinase is necessary for semaphorin 3A-induced growth cone collapse. Nat. Neurosci. 4, 367–373. 10.1038/86011 11276226

[B2] Al Moustafa A. E. Yen L. Benlimame N. Alaoui-Jamali M. A. (2002). Regulation of E-cadherin/catenin complex patterns by epidermal growth factor receptor modulation in human lung cancer cells. Lung Cancer 37, 49–56. 10.1016/S0169-5002(02)00025-9 12057867

[B3] Amano M. Ito M. Kimura K. Fukata Y. Chihara K. Nakano T.. (1996). Phosphorylation and activation of myosin by Rho-associated kinase (Rho-kinase). J. Biol. Chem. 271, 20246–20249. 8702756 10.1074/jbc.271.34.20246

[B4] Anderson S. A. Eisenstat D. D. Shi L. Rubenstein J. L. (1997). Interneuron migration from basal forebrain to neocortex: dependence on Dlx genes. Science 278, 474–476. 9334308 10.1126/science.278.5337.474

[B5] Andrews W. Liapi A. Plachez C. Camurri L. Zhang J. Mori S.. (2006). Robo1 regulates the development of major axon tracts and interneuron migration in the forebrain. Development 133, 2243–2252. 10.1242/dev.02379 16690755

[B6] Antypa M. Faux C. Eichele G. Parnavelas J. G. Andrews W. D. (2011). Differential gene expression in migratory streams of cortical interneurons. Eur. J. Neurosci. 34, 1584–1594. 10.1111/j.1460-9568.2011.07896.x 22103416 PMC3401901

[B7] Aoto K. Nishimura T. Eto K. Motoyama J. (2002). Mouse GLI3 regulates Fgf8 expression and apoptosis in the developing neural tube, face, and limb bud. Dev. Biol. 251, 320–332. 10.1006/dbio.2002.0811 12435361

[B8] Arai M. Yamada K. Toyota T. Obata N. Haga S. Yoshida Y.. (2006). Association between polymorphisms in the promoter region of the sialyltransferase 8B (SIAT8B) gene and schizophrenia. Biol. Psychiatry 59, 652–659. 10.1016/j.biopsych.2005.08.016 16229822

[B9] Avila A. Vidal P. M. Dear T. N. Harvey R. J. Rigo J. M. Nguyen L. (2013). Glycine receptor alpha2 subunit activation promotes cortical interneuron migration. Cell Rep. 4, 738–750. 10.1016/j.celrep.2013.07.016 23954789 PMC3763372

[B10] Azim E. Jabaudon D. Fame R. M. Macklis J. D. (2009). SOX6 controls dorsal progenitor identity and interneuron diversity during neocortical development. Nat. Neurosci. 12, 1238–1247. 10.1038/nn.2387 19657336 PMC2903203

[B11] Bagri A. Marin O. Plump A. S. Mak J. Pleasure S. J. Rubenstein J. L.. (2002). Slit proteins prevent midline crossing and determine the dorsoventral position of major axonal pathways in the mammalian forebrain. Neuron 33, 233–248. 10.1016/S0896-6273(02)00561-5 11804571

[B12] Bai C. B. Stephen D. Joyner A. L. (2004). All mouse ventral spinal cord patterning by hedgehog is Gli dependent and involves an activator function of Gli3. Dev. Cell 6, 103–115. 10.1016/S1534-5807(03)00394-0 14723851

[B13] Batista-Brito R. Rossignol E. Hjerling-Leffler J. Denaxa M. Wegner M. Lefebvre V.. (2009). The cell-intrinsic requirement of Sox6 for cortical interneuron development. Neuron 63, 466–481. 10.1016/j.neuron.2009.08.005 19709629 PMC2773208

[B14] Baudoin J. P. Alvarez C. Gaspar P. Metin C. (2008). Nocodazole-induced changes in microtubule dynamics impair the morphology and directionality of migrating medial ganglionic eminence cells. Dev. Neurosci. 30, 132–143. 10.1159/000109858 18075261

[B15] Baudoin J. P. Viou L. Launay P. S. Luccardini C. Espeso Gil S. Kiyasova V.. (2012). Tangentially migrating neurons assemble a primary cilium that promotes their reorientation to the cortical plate. Neuron 76, 1108–1122. 10.1016/j.neuron.2012.10.027 23259947

[B16] Behrens J. (1999). Cadherins and catenins: role in signal transduction and tumor progression. Cancer Metastasis Rev. 18, 15–30. 10505543 10.1023/a:1006200102166

[B17] Bellion A. Metin C. (2005). Early regionalisation of the neocortex and the medial ganglionic eminence. Brain Res. Bull. 66, 402–409. 10.1016/j.brainresbull.2005.07.010 16144622

[B18] Bellion A. Baudoin J. P. Alvarez C. Bornens M. Metin C. (2005). Nucleokinesis in tangentially migrating neurons comprises two alternating phases: forward migration of the Golgi/centrosome associated with centrosome splitting and myosin contraction at the rear. J. Neurosci. 25, 5691–5699. 10.1523/JNEUROSCI.1030-05.2005 15958735 PMC6724882

[B19] Bespalov M. M. Sidorova Y. A. Tumova S. Ahonen-Bishopp A. Magalhaes A. C. Kulesskiy E.. (2011). Heparan sulfate proteoglycan syndecan-3 is a novel receptor for GDNF, neurturin, and artemin. J. Cell Biol. 192, 153–169. 10.1083/jcb.201009136 21200028 PMC3019558

[B20] Besson A. Gurian-West M. Schmidt A. Hall A. Roberts J. M. (2004). p27Kip1 modulates cell migration through the regulation of RhoA activation. Genes Dev. 18, 862–876. 10.1101/gad.1185504 15078817 PMC395846

[B21] Blaess S. Corrales J. D. Joyner A. L. (2006). Sonic hedgehog regulates Gli activator and repressor functions with spatial and temporal precision in the mid/hindbrain region. Development 133, 1799–1809. 10.1242/dev.02339 16571630

[B22] Borello U. Cobos I. Long J. E. McWhirter J. R. Murre C. Rubenstein J. L. (2008). FGF15 promotes neurogenesis and opposes FGF8 function during neocortical development. Neural Dev. 3:17. 10.1186/1749-8104-3-17 18625063 PMC2492847

[B23] Bortone D. Polleux F. (2009). KCC2 expression promotes the termination of cortical interneuron migration in a voltage-sensitive calcium-dependent manner. Neuron 62, 53–71. 10.1016/j.neuron.2009.01.034 19376067 PMC3314167

[B24] Braz J. M. Sharif-Naeini R. Vogt D. Kriegstein A. Alvarez-Buylla A. Rubenstein J. L.. (2012). Forebrain GABAergic neuron precursors integrate into adult spinal cord and reduce injury-induced neuropathic pain. Neuron 74, 663–675. 10.1016/j.neuron.2012.02.033 22632725 PMC3361692

[B25] Briscoe J. Ericson J. (2001). Specification of neuronal fates in the ventral neural tube. Curr. Opin. Neurobiol. 11, 43–49. 10.1016/S0959-4388(00)00172-0 11179871

[B26] Britto J. M. Johnston L. A. Tan S. S. (2009). The stochastic search dynamics of interneuron migration. Biophys. J. 97, 699–709. 10.1016/j.bpj.2009.04.064 19651028 PMC2718142

[B27] Brown K. N. Chen S. Han Z. Lu C. H. Tan X. Zhang X. J.. (2011). Clonal production and organization of inhibitory interneurons in the neocortex. Science 334, 480–486. 10.1126/science.1208884 22034427 PMC3304494

[B28] Burke R. Nellen D. Bellotto M. Hafen E. Senti K. A. Dickson B. J.. (1999). Dispatched, a novel sterol-sensing domain protein dedicated to the release of cholesterol-modified hedgehog from signaling cells. Cell 99, 803–815. 10619433 10.1016/s0092-8674(00)81677-3

[B29] Butt S. J. Fuccillo M. Nery S. Noctor S. Kriegstein A. Corbin J. G.. (2005). The temporal and spatial origins of cortical interneurons predict their physiological subtype. Neuron 48, 591–604. 10.1016/j.neuron.2005.09.034 16301176

[B30] Butt S. J. Sousa V. H. Fuccillo M. V. Hjerling-Leffler J. Miyoshi G. Kimura S.. (2008). The requirement of Nkx2-1 in the temporal specification of cortical interneuron subtypes. Neuron 59, 722–732. 10.1016/j.neuron.2008.07.031 18786356 PMC2562525

[B31] Cai Y. Zhang Q. Wang C. Zhang Y. Ma T. Zhou X.. (2013). Nuclear receptor COUP-TFII-expressing neocortical interneurons are derived from the medial and lateral/caudal ganglionic eminence and define specific subsets of mature interneurons. J. Comp. Neurol. 521, 479–497. 10.1002/cne.23186 22791192

[B32] Cameron H. A. Hazel T. G. McKay R. D. (1998). Regulation of neurogenesis by growth factors and neurotransmitters. J. Neurobiol. 36, 287–306. 9712310

[B33] Canty A. J. Dietze J. Harvey M. Enomoto H. Milbrandt J. Ibanez C. F. (2009). Regionalized loss of parvalbumin interneurons in the cerebral cortex of mice with deficits in GFRalpha1 signaling. J. Neurosci. 29, 10695–10705. 10.1523/JNEUROSCI.2658-09.2009 19710321 PMC6665705

[B34] Casarosa S. Fode C. Guillemot F. (1999). Mash1 regulates neurogenesis in the ventral telencephalon. Development 126, 525–534. 9876181 10.1242/dev.126.3.525

[B35] Causeret F. Hidalgo-Sanchez M. Fort P. Backer S. Popoff M. R. Gauthier-Rouviere C.. (2004). Distinct roles of Rac1/Cdc42 and Rho/Rock for axon outgrowth and nucleokinesis of precerebellar neurons toward netrin 1. Development 131, 2841–2852. 10.1242/dev.01162 15151987

[B36] Chai X. Forster E. Zhao S. Bock H. H. Frotscher M. (2009a). Reelin acts as a stop signal for radially migrating neurons by inducing phosphorylation of n-cofilin at the leading edge. Commun. Integr. Biol. 2, 375–377. 10.4161/cib.2.4.8614 19721896 PMC2734053

[B37] Chai X. Forster E. Zhao S. Bock H. H. Frotscher M. (2009b). Reelin stabilizes the actin cytoskeleton of neuronal processes by inducing n-cofilin phosphorylation at serine3. J. Neurosci. 29, 288–299. 10.1523/JNEUROSCI.2934-08.2009 19129405 PMC6664910

[B38] Chrzanowska-Wodnicka M. Burridge K. (1996). Rho-stimulated contractility drives the formation of stress fibers and focal adhesions. J. Cell Biol. 133, 1403–1415. 8682874 10.1083/jcb.133.6.1403 PMC2120895

[B39] Ciceri G. Dehorter N. Sols I. Huang Z. J. Maravall M. Marin O. (2013). Lineage-specific laminar organization of cortical GABAergic interneurons. Nat. Neurosci. 16, 1199–1210. 10.1038/nn.3485 23933753

[B40] Cobos I. Calcagnotto M. E. Vilaythong A. J. Thwin M. T. Noebels J. L. Baraban S. C.. (2005). Mice lacking Dlx1 show subtype-specific loss of interneurons, reduced inhibition and epilepsy. Nat. Neurosci. 8, 1059–1068. 10.1038/nn1499 16007083

[B41] Colombo E. Collombat P. Colasante G. Bianchi M. Long J. Mansouri A.. (2007). Inactivation of Arx, the murine ortholog of the X-linked lissencephaly with ambiguous genitalia gene, leads to severe disorganization of the ventral telencephalon with impaired neuronal migration and differentiation. J. Neurosci. 27, 4786–4798. 10.1523/JNEUROSCI.0417-07.2007 17460091 PMC4916654

[B42] Corvin A. Nangle J. M. Gill M. (2004). Schizophrenia susceptibility genes: recent discoveries and new challenges. Ir. Med. J. 97, 70–72. 15164685

[B43] Cuzon V. C. Yeh P. W. Cheng Q. Yeh H. H. (2006). Ambient GABA promotes cortical entry of tangentially migrating cells derived from the medial ganglionic eminence. Cereb. Cortex 16, 1377–1388. 10.1093/cercor/bhj084 16339085

[B44] Daniel D. Rossel M. Seki T. Konig N. (2005). Stromal cell-derived factor-1 (SDF-1) expression in embryonic mouse cerebral cortex starts in the intermediate zone close to the pallial-subpallial boundary and extends progressively towards the cortical hem. Gene Expr. Patterns 5, 317–322. 10.1016/j.modgep.2004.10.007 15661637

[B45] Dantzker J. L. Callaway E. M. (2000). Laminar sources of synaptic input to cortical inhibitory interneurons and pyramidal neurons. Nat. Neurosci. 3, 701–707. 10.1038/76656 10862703

[B46] De Marco Garcia N. V. Karayannis T. Fishell G. (2011). Neuronal activity is required for the development of specific cortical interneuron subtypes. Nature 472, 351–355. 10.1038/nature09865 21460837 PMC3641515

[B47] Denaxa M. Chan C. H. Schachner M. Parnavelas J. G. Karagogeos D. (2001). The adhesion molecule TAG-1 mediates the migration of cortical interneurons from the ganglionic eminence along the corticofugal fiber system. Development 128, 4635–4644. 11714688 10.1242/dev.128.22.4635

[B48] Denaxa M. Kyriakopoulou K. Theodorakis K. Trichas G. Vidaki M. Takeda Y.. (2005). The adhesion molecule TAG-1 is required for proper migration of the superficial migratory stream in the medulla but not of cortical interneurons. Dev. Biol. 288, 87–99. 10.1016/j.ydbio.2005.09.021 16225856

[B49] Di Cristo G. Chattopadhyaya B. Kuhlman S. J. Fu Y. Belanger M. C. Wu C. Z.. (2007). Activity-dependent PSA expression regulates inhibitory maturation and onset of critical period plasticity. Nat. Neurosci. 10, 1569–1577. 10.1038/nn2008 18026099

[B50] Dobbertin A. Gervais A. Glowinski J. Mallat M. (2000). Activation of ionotropic glutamate receptors reduces the production of transforming growth factor-beta2 by developing neurons. Eur. J. Neurosci. 12, 4589–4593. 10.1046/j.0953-816X.2000.01354.x 11122374

[B51] Eagleson K. L. Campbell D. B. Thompson B. L. Bergman M. Y. Levitt P. (2011). The autism risk genes MET and PLAUR differentially impact cortical development. Autism Res. 4, 68–83. 10.1002/aur.172 21328570 PMC3644181

[B52] Echelard Y. Epstein D. J. St-Jacques B. Shen L. Mohler J. McMahon J. A.. (1993). Sonic hedgehog, a member of a family of putative signaling molecules, is implicated in the regulation of CNS polarity. Cell 75, 1417–1430. 7916661 10.1016/0092-8674(93)90627-3

[B53] Eisenstat D. D. Liu J. K. Mione M. Zhong W. Yu G. Anderson S. A.. (1999). DLX-1, DLX-2, and DLX-5 expression define distinct stages of basal forebrain differentiation. J. Comp. Neurol. 414, 217–237. 10516593 10.1002/(sici)1096-9861(19991115)414:2<217::aid-cne6>3.0.co;2-i

[B54] Fairen A. Cobas A. Fonseca M. (1986). Times of generation of glutamic acid decarboxylase immunoreactive neurons in mouse somatosensory cortex. J. Comp. Neurol. 251, 67–83. 10.1002/cne.902510105 3760259

[B55] Fazzari P. Paternain A. V. Valiente M. Pla R. Lujan R. Lloyd K.. (2010). Control of cortical GABA circuitry development by Nrg1 and ErbB4 signalling. Nature 464, 1376–1380. 10.1038/nature08928 20393464

[B56] Ferguson K. L. McClellan K. A. Vanderluit J. L. McIntosh W. C. Schuurmans C. Polleux F.. (2005). A cell-autonomous requirement for the cell cycle regulatory protein, Rb, in neuronal migration. EMBO J. 24, 4381–4391. 10.1038/sj.emboj.7600887 16308563 PMC1356328

[B57] Fertuzinhos S. Krsnik Z. Kawasawa Y. I. Rasin M. R. Kwan K. Y. Chen J. G.. (2009). Selective depletion of molecularly defined cortical interneurons in human holoprosencephaly with severe striatal hypoplasia. Cereb. Cortex 19, 2196–2207. 10.1093/cercor/bhp009 19234067 PMC2722430

[B58] Flames N. Long J. E. Garratt A. N. Fischer T. M. Gassmann M. Birchmeier C.. (2004). Short- and long-range attraction of cortical GABAergic interneurons by neuregulin-1. Neuron 44, 251–261. 10.1016/j.neuron.2004.09.028 15473965

[B59] Flames N. Pla R. Gelman D. M. Rubenstein J. L. Puelles L. Marin O. (2007). Delineation of multiple subpallial progenitor domains by the combinatorial expression of transcriptional codes. J. Neurosci. 27, 9682–9695. 10.1523/JNEUROSCI.2750-07.2007 17804629 PMC4916652

[B60] Flandin P. Zhao Y. Vogt D. Jeong J. Long J. Potter G.. (2011). Lhx6 and Lhx8 coordinately induce neuronal expression of Shh that controls the generation of interneuron progenitors. Neuron 70, 939–950. 10.1016/j.neuron.2011.04.020 21658586 PMC3153409

[B61] Fode C. Ma Q. Casarosa S. Ang S. L. Anderson D. J. Guillemot F. (2000). A role for neural determination genes in specifying the dorsoventral identity of telencephalic neurons. Genes Dev. 14, 67–80. 10.1101/gad.14.1.67 10640277 PMC316337

[B62] Fogarty M. Grist M. Gelman D. Marin O. Pachnis V. Kessaris N. (2007). Spatial genetic patterning of the embryonic neuroepithelium generates GABAergic interneuron diversity in the adult cortex. J. Neurosci. 27, 10935–10946. 10.1523/JNEUROSCI.1629-07.2007 17928435 PMC6672847

[B63] Fragkouli A. Hearn C. Errington M. Cooke S. Grigoriou M. Bliss T. (2005). Loss of forebrain cholinergic neurons and impairment in spatial learning and memory in LHX7-deficient mice. Eur. J. Neurosci. 21, 2923–2938. 10.1111/j.1460-9568.2005.04141.x 15978004

[B64] Franco S. J. Huttenlocher A. (2005). Regulating cell migration: calpains make the cut. J. Cell Sci. 118, 3829–3838. 10.1242/jcs.02562 16129881

[B65] Friocourt G. Liu J. S. Antypa M. Rakic S. Walsh C. A. Parnavelas J. G. (2007). Both doublecortin and doublecortin-like kinase play a role in cortical interneuron migration. J. Neurosci. 27, 3875–3883. 10.1523/JNEUROSCI.4530-06.2007 17409252 PMC6672408

[B66] Fuccillo M. Rallu M. McMahon A. P. Fishell G. (2004). Temporal requirement for hedgehog signaling in ventral telencephalic patterning. Development 131, 5031–5040. 10.1242/dev.01349 15371303

[B67] Gallagher P. J. Herring B. P. Stull J. T. (1997). Myosin light chain kinases. J. Muscle Res. Cell Motil. 18, 1–16. 9147985 10.1023/a:1018616814417

[B68] Gelman D. M. Marin O. (2010). Generation of interneuron diversity in the mouse cerebral cortex. Eur. J. Neurosci. 31, 2136–2141. 10.1111/j.1460-9568.2010.07267.x 20529125

[B69] Gelman D. M. Martini F. J. Nobrega-Pereira S. Pierani A. Kessaris N. Marin O. (2009). The embryonic preoptic area is a novel source of cortical GABAergic interneurons. J. Neurosci. 29, 9380–9389. 10.1523/JNEUROSCI.0604-09.2009 19625528 PMC6665570

[B70] Gelman D. Griveau A. Dehorter N. Teissier A. Varela C. Pla R.. (2011). A wide diversity of cortical GABAergic interneurons derives from the embryonic preoptic area. J. Neurosci. 31, 16570–16580. 10.1523/JNEUROSCI.4068-11.2011 22090484 PMC6633309

[B71] Giannone G. Mege R. M. Thoumine O. (2009). Multi-level molecular clutches in motile cell processes. Trends Cell Biol. 19, 475–486. 10.1016/j.tcb.2009.07.001 19716305

[B72] Glickstein S. B. Alexander S. Ross M. E. (2007a). Differences in cyclin D2 and D1 protein expression distinguish forebrain progenitor subsets. Cereb. Cortex 17, 632–642. 10.1093/cercor/bhk008 16627858

[B73] Glickstein S. B. Moore H. Slowinska B. Racchumi J. Suh M. Chuhma N.. (2007b). Selective cortical interneuron and GABA deficits in cyclin D2-null mice. Development 134, 4083–4093. 10.1242/dev.008524 17965053 PMC3396210

[B74] Glickstein S. B. Monaghan J. A. Koeller H. B. Jones T. K. Ross M. E. (2009). Cyclin D2 is critical for intermediate progenitor cell proliferation in the embryonic cortex. J. Neurosci. 29, 9614–9624. 10.1523/JNEUROSCI.2284-09.2009 19641124 PMC2811167

[B75] Godin J. D. Thomas N. Laguesse S. Malinouskaya L. Close P. Malaise O.. (2012). p27(Kip1) is a microtubule-associated protein that promotes microtubule polymerization during neuron migration. Dev. Cell 23, 729–744. 10.1016/j.devcel.2012.08.006 23022035

[B76] Gopal P. P. Simonet J. C. Shapiro W. Golden J. A. (2010). Leading process branch instability in Lis1+/- nonradially migrating interneurons. Cereb. Cortex 20, 1497–1505. 10.1093/cercor/bhp211 19861636 PMC2871376

[B77] Govek E. E. Hatten M. E. Van Aelst L. (2011). The role of Rho GTPase proteins in CNS neuronal migration. Dev. Neurobiol. 71, 528–553. 10.1002/dneu.20850 21557504 PMC3188326

[B78] Gutin G. Fernandes M. Palazzolo L. Paek H. Yu K. Ornitz D. M.. (2006). FGF signalling generates ventral telencephalic cells independently of SHH. Development 133, 2937–2946. 10.1242/dev.02465 16818446

[B79] Hansen D. V. Lui J. H. Flandin P. Yoshikawa K. Rubenstein J. L. Alvarez-Buylla A.. (2013). Non-epithelial stem cells and cortical interneuron production in the human ganglionic eminences. Nat. Neurosci. 16, 1576–1587. 10.1038/nn.3541 24097039 PMC4191718

[B80] Haydar T. F. Wang F. Schwartz M. L. Rakic P. (2000). Differential modulation of proliferation in the neocortical ventricular and subventricular zones. J. Neurosci. 20, 5764–5774. 10908617 10.1523/JNEUROSCI.20-15-05764.2000 PMC3823557

[B81] Hazan R. B. Norton L. (1998). The epidermal growth factor receptor modulates the interaction of E-cadherin with the actin cytoskeleton. J. Biol. Chem. 273, 9078–9084. 9535896 10.1074/jbc.273.15.9078

[B82] He M. Zhang Z. H. Guan C. B. Xia D. Yuan X. B. (2010). Leading tip drives soma translocation via forward F-actin flow during neuronal migration. J. Neurosci. 30, 10885–10898. 10.1523/JNEUROSCI.0240-10.2010 20702717 PMC6634710

[B83] Heasman S. J. Ridley A. J. (2008). Mammalian Rho GTPases: new insights into their functions from *in vivo* studies. Nat. Rev. Mol. Cell Biol. 9, 690–701. 10.1038/nrm2476 18719708

[B84] Hebert J. M. Fishell G. (2008). The genetics of early telencephalon patterning: some assembly required. Nat. Rev. Neurosci. 9, 678–685. 10.1038/nrn2463 19143049 PMC2669317

[B85] Heng J. I. Moonen G. Nguyen L. (2007). Neurotransmitters regulate cell migration in the telencephalon. Eur. J. Neurosci. 26, 537–546. 10.1111/j.1460-9568.2007.05694.x 17686035

[B86] Hernandez-Miranda L. R. Cariboni A. Faux C. Ruhrberg C. Cho J. H. Cloutier J. F.. (2011). Robo1 regulates semaphorin signaling to guide the migration of cortical interneurons through the ventral forebrain. J. Neurosci. 31, 6174–6187. 10.1523/JNEUROSCI.5464-10.2011 21508241 PMC3088089

[B87] Hevner R. F. Daza R. A. Englund C. Kohtz J. Fink A. (2004). Postnatal shifts of interneuron position in the neocortex of normal and reeler mice: evidence for inward radial migration. Neuroscience 124, 605–618. 10.1016/j.neuroscience.2003.11.033 14980731

[B88] Higashida C. Miyoshi T. Fujita A. Oceguera-Yanez F. Monypenny J. Andou Y.. (2004). Actin polymerization-driven molecular movement of mDia1 in living cells. Science 303, 2007–2010. 10.1126/science.1093923 15044801

[B89] Higginbotham H. R. Gleeson J. G. (2007). The centrosome in neuronal development. Trends Neurosci. 30, 276–283. 10.1016/j.tins.2007.04.001 17420058

[B90] Horton S. Meredith A. Richardson J. A. Johnson J. E. (1999). Correct coordination of neuronal differentiation events in ventral forebrain requires the bHLH factor MASH1. Mol. Cell. Neurosci. 14, 355–369. 10.1006/mcne.1999.0791 10588390

[B91] Huang C. Ni Y. Wang T. Gao Y. Haudenschild C. C. Zhan X. (1997). Down-regulation of the filamentous actin cross-linking activity of cortactin by Src-mediated tyrosine phosphorylation. J. Biol. Chem. 272, 13911–13915. 9153252 10.1074/jbc.272.21.13911

[B92] Inamura N. Kimura T. Tada S. Kurahashi T. Yanagida M. Yanagawa Y.. (2012). Intrinsic and extrinsic mechanisms control the termination of cortical interneuron migration. J. Neurosci. 32, 6032–6042. 10.1523/JNEUROSCI.3446-11.2012 22539863 PMC6703612

[B93] Ingham P. W. McMahon A. (2001). P. Hedgehog signaling in animal development: paradigms and principles. Genes Dev. 15, 3059–3087. 10.1101/gad.938601 11731473

[B94] Ishizaki T. Naito M. Fujisawa K. Maekawa M. Watanabe N. Saito Y.. (1997). p160ROCK, a Rho-associated coiled-coil forming protein kinase, works downstream of Rho and induces focal adhesions. FEBS Lett. 404, 118–124. 9119047 10.1016/s0014-5793(97)00107-5

[B95] Isomura R. Kitajima K. Sato C. (2011). Structural and functional impairments of polysialic acid by a mutated polysialyltransferase found in schizophrenia. J. Biol. Chem. 286, 21535–21545. 10.1074/jbc.M111.221143 21464126 PMC3122212

[B96] Jessell T. M. (2000). Neuronal specification in the spinal cord: inductive signals and transcriptional codes. Nat. Rev. Genet. 1, 20–29. 10.1038/35049541 11262869

[B97] Jones K. R. Farinas I. Backus C. Reichardt L. F. (1994). Targeted disruption of the BDNF gene perturbs brain and sensory neuron development but not motor neuron development. Cell 76, 989–999. 8137432 10.1016/0092-8674(94)90377-8 PMC2711896

[B98] Kappeler C. Saillour Y. Baudoin J. P. Tuy F. P. Alvarez C. Houbron C.. (2006). Branching and nucleokinesis defects in migrating interneurons derived from doublecortin knockout mice. Hum. Mol. Genet. 15, 1387–1400. 10.1093/hmg/ddl062 16571605

[B99] Kawauchi T. Chihama K. Nabeshima Y. Hoshino M. (2006). Cdk5 phosphorylates and stabilizes p27kip1 contributing to actin organization and cortical neuronal migration. Nat. Cell Biol. 8, 17–26. 10.1038/ncb1338 16341208

[B100] Kenney A. M. Rowitch D. H. (2000). Sonic hedgehog promotes G(1) cyclin expression and sustained cell cycle progression in mammalian neuronal precursors. Mol. Cell. Biol. 20, 9055–9067. 10.1128/MCB.20.23.9055-9067.2000 11074003 PMC86558

[B101] Kenney A. M. Cole M. D. Rowitch D. H. (2003). Nmyc upregulation by sonic hedgehog signaling promotes proliferation in developing cerebellar granule neuron precursors. Development 130, 15–28. 10.1242/dev.00182 12441288

[B102] Kenney A. M. Widlund H. R. Rowitch D. H. (2004). Hedgehog and PI-3 kinase signaling converge on Nmyc1 to promote cell cycle progression in cerebellar neuronal precursors. Development 131, 217–228. 10.1242/dev.00891 14660435

[B103] Kepecs A. Fishell G. (2014). Interneuron cell types are fit to function. Nature 505, 318–326. 10.1038/nature12983 24429630 PMC4349583

[B104] Kimura K. Ito M. Amano M. Chihara K. Fukata Y. Nakafuku M.. (1996). Regulation of myosin phosphatase by Rho and Rho-associated kinase (Rho-kinase). Science 273, 245–248. 8662509 10.1126/science.273.5272.245

[B105] Kohtz J. D. Lee H. Y. Gaiano N. Segal J. Ng E. Larson T.. (2001). N-terminal fatty-acylation of sonic hedgehog enhances the induction of rodent ventral forebrain neurons. Development 128, 2351–2363. 11493554 10.1242/dev.128.12.2351

[B106] Kullander K. Klein R. (2002). Mechanisms and functions of Eph and ephrin signalling. Nat. Rev. Mol. Cell Biol. 3, 475–486. 10.1038/nrm856 12094214

[B107] Kumada T. Komuro H. (2004). Completion of neuronal migration regulated by loss of Ca(2+) transients. Proc. Natl. Acad. Sci. U.S.A. 101, 8479–8484. 10.1073/pnas.0401000101 15150416 PMC420419

[B108] Kuschel S. Ruther U. Theil T. (2003). A disrupted balance between Bmp/Wnt and Fgf signaling underlies the ventralization of the Gli3 mutant telencephalon. Dev. Biol. 260, 484–495. 10.1016/S0012-1606(03)00252-5 12921747

[B109] Laurie D. J. Wisden W. Seeburg P. H. (1992). The distribution of thirteen GABAA receptor subunit mRNAs in the rat brain. III. Embryonic and postnatal development. J. Neurosci. 12, 4151–4172. 1331359 10.1523/JNEUROSCI.12-11-04151.1992 PMC6576006

[B110] Lavdas A. A. Grigoriou M. Pachnis V. Parnavelas J. G. (1999). The medial ganglionic eminence gives rise to a population of early neurons in the developing cerebral cortex. J. Neurosci. 19, 7881–7888. 10479690 10.1523/JNEUROSCI.19-18-07881.1999 PMC6782477

[B111] Li G. Adesnik H. Li J. Long J. Nicoll R. A. Rubenstein J. L.. (2008). Regional distribution of cortical interneurons and development of inhibitory tone are regulated by Cxcl12/Cxcr4 signaling. J. Neurosci. 28, 1085–1098. 10.1523/JNEUROSCI.4602-07.2008 18234887 PMC3072297

[B112] Liodis P. Denaxa M. Grigoriou M. Akufo-Addo C. Yanagawa Y. Pachnis V. (2007). Lhx6 activity is required for the normal migration and specification of cortical interneuron subtypes. J. Neurosci. 27, 3078–3089. 10.1523/JNEUROSCI.3055-06.2007 17376969 PMC6672459

[B113] Liu J. K. Ghattas I. Liu S. Chen S. Rubenstein J. L. (1997). Dlx genes encode DNA-binding proteins that are expressed in an overlapping and sequential pattern during basal ganglia differentiation. Dev. Dyn. 210, 498–512. 10.1002/(SICI)1097-0177(199712)210:4<498::AID-AJA12>3.0.CO;2-3 9415433

[B114] Lodato S. Tomassy G. S. De Leonibus E. Uzcategui Y. G. Andolfi G. Armentano M.. (2011a). Loss of COUP-TFI alters the balance between caudal ganglionic eminence- and medial ganglionic eminence-derived cortical interneurons and results in resistance to epilepsy. J. Neurosci. 31, 4650–4662. 10.1523/JNEUROSCI.6580-10.2011 21430164 PMC6622915

[B115] Lodato S. Rouaux C. Quast K. B. Jantrachotechatchawan C. Studer M. Hensch T. K.. (2011b). Excitatory projection neuron subtypes control the distribution of local inhibitory interneurons in the cerebral cortex. Neuron 69, 763–779. 10.1016/j.neuron.2011.01.015 21338885 PMC3061282

[B116] Long J. E. Cobos I. Potter G. B. Rubenstein J. L. (2009a). Dlx1&2 and Mash1 transcription factors control MGE and CGE patterning and differentiation through parallel and overlapping pathways. Cereb. Cortex 19(Suppl. 1), i96–i106. 10.1093/cercor/bhp045 19386638 PMC2693539

[B117] Long J. E. Swan C. Liang W. S. Cobos I. Potter G. B. Rubenstein J. L. (2009b). Dlx1&2 and Mash1 transcription factors control striatal patterning and differentiation through parallel and overlapping pathways. J. Comp. Neurol. 512, 556–572. 10.1002/cne.21854 19030180 PMC2761428

[B118] Lopez-Bendito G. Lujan R. Shigemoto R. Ganter P. Paulsen O. Molnar Z. (2003). Blockade of GABA(B) receptors alters the tangential migration of cortical neurons. Cereb. Cortex 13, 932–942. 10.1093/cercor/13.9.932 12902392

[B119] Lopez-Bendito G. Sanchez-Alcaniz J. A. Pla R. Borrell V. Pico E. Valdeolmillos M.. (2008). Chemokine signaling controls intracortical migration and final distribution of GABAergic interneurons. J. Neurosci. 28, 1613–1624. 10.1523/JNEUROSCI.4651-07.2008 18272682 PMC6671533

[B120] LoTurco J. J. Owens D. F. Heath M. J. Davis M. B. Kriegstein A. R. (1995). GABA and glutamate depolarize cortical progenitor cells and inhibit DNA synthesis. Neuron 15, 1287–1298. 8845153 10.1016/0896-6273(95)90008-x

[B121] Luccardini C. Hennekinne L. Viou L. Yanagida M. Murakami F. Kessaris N.. (2013). N-cadherin sustains motility and polarity of future cortical interneurons during tangential migration. J. Neurosci. 33, 18149–18160. 10.1523/JNEUROSCI.0593-13.2013 24227724 PMC3858641

[B122] Luk K. C. Kennedy T. E. Sadikot A. F. (2003). Glutamate promotes proliferation of striatal neuronal progenitors by an NMDA receptor-mediated mechanism. J. Neurosci. 23, 2239–2250. 12657683 10.1523/JNEUROSCI.23-06-02239.2003 PMC6742023

[B123] Lukaszewicz A. Savatier P. Cortay V. Kennedy H. Dehay C. (2002). Contrasting effects of basic fibroblast growth factor and neurotrophin 3 on cell cycle kinetics of mouse cortical stem cells. J. Neurosci. 22, 6610–6622. 12151540 10.1523/JNEUROSCI.22-15-06610.2002 PMC2001296

[B124] Lyons M. R. Schwarz C. M. West A. E. (2012). Members of the myocyte enhancer factor 2 transcription factor family differentially regulate Bdnf transcription in response to neuronal depolarization. J. Neurosci. 32, 12780–12785. 10.1523/JNEUROSCI.0534-12.2012 22973001 PMC3487695

[B125] Lysko D. E. Putt M. Golden J. A. (2014). SDF1 reduces interneuron leading process branching through dual regulation of actin and microtubules. J. Neurosci. 34, 4941–4962. 10.1523/JNEUROSCI.4351-12.2014 24695713 PMC3972721

[B126] Ma W. Barker J. L. (1995). Complementary expressions of transcripts encoding GAD67 and GABAA receptor alpha 4, beta 1, and gamma 1 subunits in the proliferative zone of the embryonic rat central nervous system. J. Neurosci. 15, 2547–2560. 7891188 10.1523/JNEUROSCI.15-03-02547.1995 PMC6578170

[B127] Ma T. Zhang Q. Cai Y. You Y. Rubenstein J. L. Yang Z. (2012). A subpopulation of dorsal lateral/caudal ganglionic eminence-derived neocortical interneurons expresses the transcription factor Sp8. Cereb. Cortex 22, 2120–2130. 10.1093/cercor/bhr296 22021915

[B128] Ma T. Wang C. Wang L. Zhou X. Tian M. Zhang Q.. (2013). Subcortical origins of human and monkey neocortical interneurons. Nat. Neurosci. 16, 1588–1597. 10.1038/nn.3536 24097041

[B129] Manent J. B. Jorquera I. Ben-Ari Y. Aniksztejn L. Represa A. (2006). Glutamate acting on AMPA but not NMDA receptors modulates the migration of hippocampal interneurons. J. Neurosci. 26, 5901–5909. 10.1523/JNEUROSCI.1033-06.2006 16738232 PMC6675217

[B130] Maric D. Liu Q. Y. Maric I. Chaudry S. Chang Y. H. Smith S. V.. (2001). GABA expression dominates neuronal lineage progression in the embryonic rat neocortex and facilitates neurite outgrowth via GABA(A) autoreceptor/Cl- channels. J. Neurosci. 21, 2343–2360. 11264309 10.1523/JNEUROSCI.21-07-02343.2001 PMC6762405

[B131] Marillat V. Cases O. Nguyen-Ba-Charvet K. T. Tessier-Lavigne M. Sotelo C. Chedotal A. (2002). Spatiotemporal expression patterns of slit and robo genes in the rat brain. J. Comp. Neurol. 442, 130–155. 10.1002/cne.10068 11754167

[B132] Marin O. (2012). Interneuron dysfunction in psychiatric disorders. Nat. Rev. Neurosci. 13, 107–120. 10.1038/nrn3155 22251963

[B133] Marin O. Anderson S. A. Rubenstein J. L. (2000). Origin and molecular specification of striatal interneurons. J. Neurosci. 20, 6063–6076. 10934256 10.1523/JNEUROSCI.20-16-06063.2000 PMC6772576

[B134] Marin O. Yaron A. Bagri A. Tessier-Lavigne M. Rubenstein J. L. (2001). Sorting of striatal and cortical interneurons regulated by semaphorin-neuropilin interactions. Science 293, 872–875. 10.1126/science.1061891 11486090

[B135] Marin O. Baker J. Puelles L. Rubenstein J. L. (2002). Patterning of the basal telencephalon and hypothalamus is essential for guidance of cortical projections. Development 129, 761–773. 11830575 10.1242/dev.129.3.761

[B136] Marin O. Plump A. S. Flames N. Sanchez-Camacho C. Tessier-Lavigne M. Rubenstein J. L. (2003). Directional guidance of interneuron migration to the cerebral cortex relies on subcortical Slit1/2-independent repulsion and cortical attraction. Development 130, 1889–1901. 10.1242/dev.00417 12642493

[B137] Martinez-Cerdeno V. Noctor S. C. Espinosa A. Ariza J. Parker P. Orasji S.. (2010). Embryonic MGE precursor cells grafted into adult rat striatum integrate and ameliorate motor symptoms in 6-OHDA-lesioned rats. Cell Stem Cell 6, 238–250. 10.1016/j.stem.2010.01.004 20207227 PMC4075336

[B138] Martini F. J. Valdeolmillos M. (2010). Actomyosin contraction at the cell rear drives nuclear translocation in migrating cortical interneurons. J. Neurosci. 30, 8660–8670. 10.1523/JNEUROSCI.1962-10.2010 20573911 PMC6634617

[B139] Martini F. J. Valiente M. Lopez Bendito G. Szabo G. Moya F. Valdeolmillos M.. (2009). Biased selection of leading process branches mediates chemotaxis during tangential neuronal migration. Development 136, 41–50. 10.1242/dev.025502 19060332

[B140] Martynoga B. Morrison H. Price D. J. Mason J. O. (2005). Foxg1 is required for specification of ventral telencephalon and region-specific regulation of dorsal telencephalic precursor proliferation and apoptosis. Dev. Biol. 283, 113–127. 10.1016/j.ydbio.2005.04.005 15893304

[B141] McKinsey G. L. Lindtner S. Trzcinski B. Visel A. Pennacchio L. A. Huylebroeck D.. (2013). Dlx1&2-dependent expression of Zfhx1b (Sip1, Zeb2) regulates the fate switch between cortical and striatal interneurons. Neuron 77, 83–98. 10.1016/j.neuron.2012.11.035 23312518 PMC3547499

[B142] McManus M. F. Nasrallah I. M. Pancoast M. M. Wynshaw-Boris A. Golden J. A. (2004). Lis1 is necessary for normal non-radial migration of inhibitory interneurons. Am. J. Pathol. 165, 775–784. 10.1016/S0002-9440(10)63340-8 15331402 PMC2336110

[B143] Metin C. Godement P. (1996). The ganglionic eminence may be an intermediate target for corticofugal and thalamocortical axons. J. Neurosci. 16, 3219–3235. 8627360 10.1523/JNEUROSCI.16-10-03219.1996 PMC6579142

[B144] Metin C. Denizot J. P. Ropert N. (2000). Intermediate zone cells express calcium-permeable AMPA receptors and establish close contact with growing axons. J. Neurosci. 20, 696–708. 10632599 10.1523/JNEUROSCI.20-02-00696.2000 PMC6772402

[B145] Metin C. Baudoin J. P. Rakic S. Parnavelas J. G. (2006). Cell and molecular mechanisms involved in the migration of cortical interneurons. Eur. J. Neurosci. 23, 894–900. 10.1111/j.1460-9568.2006.04630.x 16519654

[B146] Millar J. K. Christie S. Semple C. A. Porteous D. J. (2000a). Chromosomal location and genomic structure of the human translin-associated factor X gene (TRAX; TSNAX) revealed by intergenic splicing to DISC1, a gene disrupted by a translocation segregating with schizophrenia. Genomics 67, 69–77. 10.1006/geno.2000.6239 10945471

[B147] Millar J. K. Wilson-Annan J. C. Anderson S. Christie S. Taylor M. S. Semple C. A.. (2000b). Disruption of two novel genes by a translocation co-segregating with schizophrenia. Hum. Mol. Genet. 9, 1415–1423. 10.1093/hmg/9.9.1415 10814723

[B148] Miller M. W. (1985). Cogeneration of retrogradely labeled corticocortical projection and GABA-immunoreactive local circuit neurons in cerebral cortex. Brain Res. 355, 187–192. 3910166 10.1016/0165-3806(85)90040-9

[B149] Miyoshi G. Fishell G. (2011). GABAergic interneuron lineages selectively sort into specific cortical layers during early postnatal development. Cereb. Cortex 21, 845–852. 10.1093/cercor/bhq155 20732898 PMC3059886

[B150] Miyoshi G. Hjerling-Leffler J. Karayannis T. Sousa V. H. Butt S. J. Battiste J.. (2010). Genetic fate mapping reveals that the caudal ganglionic eminence produces a large and diverse population of superficial cortical interneurons. J. Neurosci. 30, 1582–1594. 10.1523/JNEUROSCI.4515-09.2010 20130169 PMC2826846

[B151] Mountcastle V. B. (1997). The columnar organization of the neocortex. Brain 120(Pt 4), 701–722. 9153131 10.1093/brain/120.4.701

[B152] Nadarajah B. Alifragis P. Wong R. O. Parnavelas J. G. (2002). Ventricle-directed migration in the developing cerebral cortex. Nat. Neurosci. 5, 218–224. 10.1038/nn813 11850632

[B153] Nasrallah I. M. McManus M. F. Pancoast M. M. Wynshaw-Boris A. Golden J. A. (2006). Analysis of non-radial interneuron migration dynamics and its disruption in Lis1+/- mice. J. Comp. Neurol. 496, 847–858. 10.1002/cne.20966 16628622

[B154] Neddens J. Buonanno A. (2010). Selective populations of hippocampal interneurons express ErbB4 and their number and distribution is altered in ErbB4 knockout mice. Hippocampus 20, 724–744. 10.1002/hipo.20675 19655320 PMC2958210

[B155] Nguyen L. Rigo J. M. Rocher V. Belachew S. Malgrange B. Rogister B.. (2001). Neurotransmitters as early signals for central nervous system development. Cell Tissue Res. 305, 187–202. 10.1007/s004410000343 11545256

[B156] Nguyen L. Besson A. Heng J. I. Schuurmans C. Teboul L. Parras C.. (2006). p27kip1 independently promotes neuronal differentiation and migration in the cerebral cortex. Genes Dev. 20, 1511–1524. 10.1101/gad.377106 16705040 PMC1475763

[B157] Ni C. Y. Murphy M. P. Golde T. E. Carpenter G. (2001). gamma -Secretase cleavage and nuclear localization of ErbB-4 receptor tyrosine kinase. Science 294, 2179–2181. 10.1126/science.1065412 11679632

[B158] Nobrega-Pereira S. Kessaris N. Du T. Kimura S. Anderson S. A. Marin O. (2008). Postmitotic Nkx2-1 controls the migration of telencephalic interneurons by direct repression of guidance receptors. Neuron 59, 733–745. 10.1016/j.neuron.2008.07.024 18786357 PMC2643060

[B159] Ohkubo Y. Chiang C. Rubenstein J. L. (2002). Coordinate regulation and synergistic actions of BMP4, SHH and FGF8 in the rostral prosencephalon regulate morphogenesis of the telencephalic and optic vesicles. Neuroscience 111, 1–17. 10.1016/S0306-4522(01)00616-9 11955708

[B160] Ohtani N. Goto T. Waeber C. Bhide P. G. (2003). Dopamine modulates cell cycle in the lateral ganglionic eminence. J. Neurosci. 23, 2840–2850. 12684471 10.1523/JNEUROSCI.23-07-02840.2003 PMC1201391

[B161] Oliver T. G. Grasfeder L. L. Carroll A. L. Kaiser C. Gillingham C. L. Lin S. M.. (2003). Transcriptional profiling of the Sonic hedgehog response: a critical role for N-myc in proliferation of neuronal precursors. Proc. Natl. Acad. Sci. U.S.A. 100, 7331–7336. 10.1073/pnas.0832317100 12777630 PMC165875

[B162] Owens D. F. Kriegstein A. R. (2002a). Is there more to GABA than synaptic inhibition? Nat. Rev. Neurosci. 3, 715–727. 10.1038/nrn919 12209120

[B163] Owens D. F. Kriegstein A. R. (2002b). Developmental neurotransmitters? Neuron 36, 989–991. 10.1016/S0896-6273(02)01136-4 12495613

[B164] Owens D. F. Liu X. Kriegstein A. R. (1999). Changing properties of GABA(A) receptor-mediated signaling during early neocortical development. J. Neurophysiol. 82, 570–583. 10444657 10.1152/jn.1999.82.2.570

[B165] Pan Y. Bai C. B. Joyner A. L. Wang B. (2006). Sonic hedgehog signaling regulates Gli2 transcriptional activity by suppressing its processing and degradation. Mol. Cell. Biol. 26, 3365–3377. 10.1128/MCB.26.9.3365-3377.2006 16611981 PMC1447407

[B166] Parras C. M. Hunt C. Sugimori M. Nakafuku M. Rowitch D. Guillemot F. (2007). The proneural gene Mash1 specifies an early population of telencephalic oligodendrocytes. J. Neurosci. 27, 4233–4242. 10.1523/JNEUROSCI.0126-07.2007 17442807 PMC6672315

[B167] Pei Z. Wang B. Chen G. Nagao M. Nakafuku M. Campbell K. (2011). Homeobox genes Gsx1 and Gsx2 differentially regulate telencephalic progenitor maturation. Proc. Natl. Acad. Sci. U.S.A. 108, 1675–1680. 10.1073/pnas.1008824108 21205889 PMC3029701

[B168] Petilla Interneuron Nomenclature G. Ascoli G. A. Alonso-Nanclares L. Anderson S. A. Barrionuevo G. Benavides-Piccione R.. (2008). Petilla terminology: nomenclature of features of GABAergic interneurons of the cerebral cortex. Nat. Rev. Neurosci. 9, 557–568. 10.1038/nrn2402 18568015 PMC2868386

[B169] Petryniak M. A. Potter G. B. Rowitch D. H. Rubenstein J. L. (2007). Dlx1 and Dlx2 control neuronal versus oligodendroglial cell fate acquisition in the developing forebrain. Neuron 55, 417–433. 10.1016/j.neuron.2007.06.036 17678855 PMC2039927

[B170] Pilz G. A. Shitamukai A. Reillo I. Pacary E. Schwausch J. Stahl R.. (2013). Amplification of progenitors in the mammalian telencephalon includes a new radial glial cell type. Nat. Commun. 4:2125. 10.1038/ncomms3125 23839311 PMC3717501

[B171] Pla R. Borrell V. Flames N. Marin O. (2006). Layer acquisition by cortical GABAergic interneurons is independent of Reelin signaling. J. Neurosci. 26, 6924–6934. 10.1523/JNEUROSCI.0245-06.2006 16807322 PMC6673924

[B172] Pleasure S. J. Anderson S. Hevner R. Bagri A. Marin O. Lowenstein D. H.. (2000). Cell migration from the ganglionic eminences is required for the development of hippocampal GABAergic interneurons. Neuron 28, 727–740. 10.1016/S0896-6273(00)00149-5 11163262

[B173] Poitras L. Ghanem N. Hatch G. Ekker M. (2007). The proneural determinant MASH1 regulates forebrain Dlx1/2 expression through the I12b intergenic enhancer. Development 134, 1755–1765. 10.1242/dev.02845 17409112

[B174] Polleux F. Whitford K. L. Dijkhuizen P. A. Vitalis T. Ghosh A. (2002). Control of cortical interneuron migration by neurotrophins and PI3-kinase signaling. Development 129, 3147–3160. 12070090 10.1242/dev.129.13.3147

[B175] Porter J. A. Young K. E. Beachy P. A. (1996). Cholesterol modification of hedgehog signaling proteins in animal development. Science 274, 255–259. 8824192 10.1126/science.274.5285.255

[B176] Powell E. M. Mars W. M. Levitt P. (2001). Hepatocyte growth factor/scatter factor is a motogen for interneurons migrating from the ventral to dorsal telencephalon. Neuron 30, 79–89. 10.1016/S0896-6273(01)00264-1 11343646

[B177] Pozas E. Ibanez C. F. (2005). GDNF and GFRalpha1 promote differentiation and tangential migration of cortical GABAergic neurons. Neuron 45, 701–713. 10.1016/j.neuron.2005.01.043 15748846

[B178] Rallu M. Machold R. Gaiano N. Corbin J. G. McMahon A. P. Fishell G. (2002). Dorsoventral patterning is established in the telencephalon of mutants lacking both Gli3 and Hedgehog signaling. Development 129, 4963–4974. 12397105 10.1242/dev.129.21.4963

[B179] Rash B. G. Grove E. A. (2007). Patterning the dorsal telencephalon: a role for sonic hedgehog? J. Neurosci. 27, 11595–11603. 10.1523/JNEUROSCI.3204-07.2007 17959802 PMC6673221

[B180] Ross M. E. (2011). Cell cycle regulation and interneuron production. Dev. Neurobiol. 71, 2–9. 10.1002/dneu.20823 21154905 PMC3288581

[B181] Rowitch D. H. S-Jacques B. Lee S. M. Flax J. D. Snyder E. Y. McMahon A. P. (1999). Sonic hedgehog regulates proliferation and inhibits differentiation of CNS precursor cells. J. Neurosci. 19, 8954–8965. 10516314 10.1523/JNEUROSCI.19-20-08954.1999 PMC6782773

[B182] Rubin A. N. Alfonsi F. Humphreys M. P. Choi C. K. Rocha S. F. Kessaris N. (2010). The germinal zones of the basal ganglia but not the septum generate GABAergic interneurons for the cortex. J. Neurosci. 30, 12050–12062. 10.1523/JNEUROSCI.6178-09.2010 20826668 PMC3044873

[B183] Ruzhynsky V. A. McClellan K. A. Vanderluit J. L. Jeong Y. Furimsky M. Park D. S.. (2007). Cell cycle regulator E2F4 is essential for the development of the ventral telencephalon. J. Neurosci. 27, 5926–5935. 10.1523/JNEUROSCI.1538-07.2007 17537963 PMC6672261

[B184] Rymar V. V. Sadikot A. F. (2007). Laminar fate of cortical GABAergic interneurons is dependent on both birthdate and phenotype. J. Comp. Neurol. 501, 369–380. 10.1002/cne.21250 17245711

[B185] Sadikot A. F. Burhan A. M. Belanger M. C. Sasseville R. (1998). NMDA receptor antagonists influence early development of GABAergic interneurons in the mammalian striatum. Brain Res. Dev. Brain Res. 105, 35–42. 9497077

[B186] Sanchez-Huertas C. Rico B. (2011). CREB-Dependent Regulation of GAD65 Transcription by BDNF/TrkB in Cortical Interneurons. Cereb. Cortex 21, 777–788. 10.1093/cercor/bhq150 20739478

[B187] Scaltriti M. Baselga J. (2006). The epidermal growth factor receptor pathway: a model for targeted therapy. Clin. Cancer Res. 12, 5268–5272. 10.1158/1078-0432.CCR-05-1554 17000658

[B188] Schaar B. T. McConnell S. K. (2005). Cytoskeletal coordination during neuronal migration. Proc. Natl. Acad. Sci. U.S.A. 102, 13652–13657. 10.1073/pnas.0506008102 16174753 PMC1199551

[B189] Schaefer A. W. Kabir N. Forscher P. (2002). Filopodia and actin arcs guide the assembly and transport of two populations of microtubules with unique dynamic parameters in neuronal growth cones. J. Cell Biol. 158, 139–152. 10.1083/jcb.200203038 12105186 PMC2173029

[B190] Sheth A. N. Bhide P. G. (1997). Concurrent cellular output from two proliferative populations in the early embryonic mouse corpus striatum. J. Comp. Neurol. 383, 220–230. 9182850

[B191] Shimamura K. Hartigan D. J. Martinez S. Puelles L. Rubenstein J. L. (1995). Longitudinal organization of the anterior neural plate and neural tube. Development 121, 3923–3933. 8575293 10.1242/dev.121.12.3923

[B192] Shinohara R. Thumkeo D. Kamijo H. Kaneko N. Sawamoto K. Watanabe K.. (2012). A role for mDia, a Rho-regulated actin nucleator, in tangential migration of interneuron precursors. Nat. Neurosci. 15, 373–380, S371–S372. 10.1038/nn.3020 22246438

[B193] Silva C. G. Metin C. Fazeli W. Machado N. J. Darmopil S. Launay P. S.. (2013). Adenosine receptor antagonists including caffeine alter fetal brain development in mice. Sci. Transl. Med. 5:197ra104. 10.1126/scitranslmed.3006258 23926202

[B194] Sjostrom S. K. Finn G. Hahn W. C. Rowitch D. H. Kenney A. M. (2005). The Cdk1 complex plays a prime role in regulating N-myc phosphorylation and turnover in neural precursors. Dev. Cell 9, 327–338. 10.1016/j.devcel.2005.07.014 16139224

[B195] Smart I. H. (1976). A pilot study of cell production by the ganglionic eminences of the developing mouse brain. J. Anat. 121, 71–84. 1254534 PMC1231820

[B196] Smith K. M. Maragnoli M. E. Phull P. M. Tran K. M. Choubey L. Vaccarino F. M. (2014). Fgfr1 inactivation in the mouse telencephalon results in impaired maturation of interneurons expressing parvalbumin. PLoS ONE 9:e103696. 10.1371/journal.pone.0103696 25116473 PMC4130531

[B197] Southwell D. G. Paredes M. F. Galvao R. P. Jones D. L. Froemke R. C. Sebe J. Y.. (2012). Intrinsically determined cell death of developing cortical interneurons. Nature 491, 109–113. 10.1038/nature11523 23041929 PMC3726009

[B198] Spillane M. Ketschek A. Jones S. L. Korobova F. Marsick B. Lanier L.. (2011). The actin nucleating Arp2/3 complex contributes to the formation of axonal filopodia and branches through the regulation of actin patch precursors to filopodia. Dev. Neurobiol. 71, 747–758. 10.1002/dneu.20907 21557512 PMC3154400

[B199] Spoelgen R. Hammes A. Anzenberger U. Zechner D. Andersen O. M. Jerchow B.. (2005). LRP2/megalin is required for patterning of the ventral telencephalon. Development 132, 405–414. 10.1242/dev.01580 15623804

[B200] Stanco A. Szekeres C. Patel N. Rao S. Campbell K. Kreidberg J. A.. (2009). Netrin-1-alpha3beta1 integrin interactions regulate the migration of interneurons through the cortical marginal zone. Proc. Natl. Acad. Sci. U.S.A. 106, 7595–7600. 10.1073/pnas.0811343106 19383784 PMC2678634

[B201] Steinecke A. Gampe C. Nitzsche F. Bolz J. (2014a). DISC1 knockdown impairs the tangential migration of cortical interneurons by affecting the actin cytoskeleton. Front. Cell. Neurosci. 8:190. 10.3389/fncel.2014.00190 25071449 PMC4086047

[B202] Steinecke A. Gampe C. Zimmer G. Rudolph J. Bolz J. (2014b). EphA/ephrin A reverse signaling promotes the migration of cortical interneurons from the medial ganglionic eminence. Development 141, 460–471. 10.1242/dev.101691 24381199

[B203] Storm E. E. Garel S. Borello U. Hebert J. M. Martinez S. McConnell S. K.. (2006). Dose-dependent functions of Fgf8 in regulating telencephalic patterning centers. Development 133, 1831–1844. 10.1242/dev.02324 16613831

[B204] Stumm R. Kolodziej A. Schulz S. Kohtz J. D. Hollt V. (2007). Patterns of SDF-1alpha and SDF-1gamma mRNAs, migration pathways, and phenotypes of CXCR4-expressing neurons in the developing rat telencephalon. J. Comp. Neurol. 502, 382–399. 10.1002/cne.21336 17366607

[B205] Sussel L. Marin O. Kimura S. Rubenstein J. L. (1999). Loss of Nkx2.1 homeobox gene function results in a ventral to dorsal molecular respecification within the basal telencephalon: evidence for a transformation of the pallidum into the striatum. Development 126, 3359–3370. 10393115 10.1242/dev.126.15.3359

[B206] Tanaka T. Serneo F. F. Higgins C. Gambello M. J. Wynshaw-Boris A. Gleeson J. G. (2004). Lis1 and doublecortin function with dynein to mediate coupling of the nucleus to the centrosome in neuronal migration. J. Cell Biol. 165, 709–721. 10.1083/jcb.200309025 15173193 PMC2172383

[B207] Tanaka D. H. Mikami S. Nagasawa T. Miyazaki J. Nakajima K. Murakami F. (2010). CXCR4 is required for proper regional and laminar distribution of cortical somatostatin-, calretinin-, and neuropeptide Y-expressing GABAergic interneurons. Cereb. Cortex 20, 2810–2817. 10.1093/cercor/bhq027 20200107

[B208] Tao R. Li C. Zheng Y. Qin W. Zhang J. Li X.. (2007). Positive association between SIAT8B and schizophrenia in the Chinese Han population. Schizophr. Res. 90, 108–114. 10.1016/j.schres.2006.09.029 17126533

[B209] Theil T. Dominguez-Frutos E. Schimmang T. (2008). Differential requirements for Fgf3 and Fgf8 during mouse forebrain development. Dev. Dyn. 237, 3417–3423. 10.1002/dvdy.21765 18942154

[B210] Thomas S. M. Brugge J. S. (1997). Cellular functions regulated by Src family kinases. Annu. Rev. Cell Dev. Biol. 13, 513–609. 10.1146/annurev.cellbio.13.1.513 9442882

[B211] Tiveron M. C. Rossel M. Moepps B. Zhang Y. L. Seidenfaden R. Favor J.. (2006). Molecular interaction between projection neuron precursors and invading interneurons via stromal-derived factor 1 (CXCL12)/CXCR4 signaling in the cortical subventricular zone/intermediate zone. J. Neurosci. 26, 13273–13278. 10.1523/JNEUROSCI.4162-06.2006 17182777 PMC6674999

[B212] Tivodar S. Kalemaki K. Kounoupa Z. Vidaki M. Theodorakis K. Denaxa M.. (2014). Rac-GTPases regulate microtubule stability and axon growth of cortical GABAergic interneurons. Cereb. Cortex. [Epub ahead of print]. 10.1093/cercor/bhu037 24626607 PMC4537417

[B213] Toritsuka M. Kimoto S. Muraki K. Landek-Salgado M. A. Yoshida A. Yamamoto N.. (2013). Deficits in microRNA-mediated Cxcr4/Cxcl12 signaling in neurodevelopmental deficits in a 22q11 deletion syndrome mouse model. Proc. Natl. Acad. Sci. U.S.A. 110, 17552–17557. 10.1073/pnas.1312661110 24101523 PMC3808586

[B214] Trimarchi J. M. Lees J. A. (2002). Sibling rivalry in the E2F family. Nat. Rev. Mol. Cell Biol. 3, 11–20. 10.1038/nrm714 11823794

[B215] Tsai J. W. Bremner K. H. Vallee R. B. (2007). Dual subcellular roles for LIS1 and dynein in radial neuronal migration in live brain tissue. Nat. Neurosci. 10, 970–979. 10.1038/nn1934 17618279

[B216] Tuncdemir S. N. Fishell G. Batista-Brito R. (2014). miRNAs are essential for the survival and maturation of cortical interneurons. Cereb. Cortex. [Epub ahead of print]. 10.1093/cercor/bht426 24451661 PMC4459287

[B217] Uchida Y. Ohshima T. Sasaki Y. Suzuki H. Yanai S. Yamashita N.. (2005). Semaphorin3A signalling is mediated via sequential Cdk5 and GSK3beta phosphorylation of CRMP2: implication of common phosphorylating mechanism underlying axon guidance and Alzheimer's disease. Genes Cells 10, 165–179. 10.1111/j.1365-2443.2005.00827.x 15676027

[B218] Valcanis H. Tan S. S. (2003). Layer specification of transplanted interneurons in developing mouse neocortex. J. Neurosci. 23, 5113–5122. 12832535 10.1523/JNEUROSCI.23-12-05113.2003 PMC6741168

[B219] Vidaki M. Tivodar S. Doulgeraki K. Tybulewicz V. Kessaris N. Pachnis V.. (2012). Rac1-dependent cell cycle exit of MGE precursors and GABAergic interneuron migration to the cortex. Cereb. Cortex 22, 680–692. 10.1093/cercor/bhr145 21690261 PMC3589917

[B220] Wahl S. Barth H. Ciossek T. Aktories K. Mueller B. K. (2000). Ephrin-A5 induces collapse of growth cones by activating Rho and Rho kinase. J. Cell Biol. 149, 263–270. 10769020 10.1083/jcb.149.2.263 PMC2175154

[B221] Wang Y. Dye C. A. Sohal V. Long J. E. Estrada R. C. Roztocil T.. (2010). Dlx5 and Dlx6 regulate the development of parvalbumin-expressing cortical interneurons. J. Neurosci. 30, 5334–5345. 10.1523/JNEUROSCI.5963-09.2010 20392955 PMC2919857

[B222] Wang Y. Li G. Stanco A. Long J. E. Crawford D. Potter G. B.. (2011). CXCR4 and CXCR7 have distinct functions in regulating interneuron migration. Neuron 69, 61–76. 10.1016/j.neuron.2010.12.005 21220099 PMC3025760

[B223] Weaver A. M. Karginov A. V. Kinley A. W. Weed S. A. Li Y. Parsons J. T.. (2001). Cortactin promotes and stabilizes Arp2/3-induced actin filament network formation. Curr. Biol. 11, 370–374. 11267876 10.1016/s0960-9822(01)00098-7

[B224] Weed S. A. Parsons J. T. (2001). Cortactin: coupling membrane dynamics to cortical actin assembly. Oncogene 20, 6418–6434. 10.1038/sj.onc.1204783 11607842

[B225] West A. E. Greenberg M. E. (2011). Neuronal activity-regulated gene transcription in synapse development and cognitive function. Cold Spring Harb Perspect Biol 3. 10.1101/cshperspect.a005744 21555405 PMC3098681

[B226] Wonders C. P. Anderson S. A. (2006). The origin and specification of cortical interneurons. Nat. Rev. Neurosci. 7, 687–696. 10.1038/nrn1954 16883309

[B227] Wonders C. P. Taylor L. Welagen J. Mbata I. C. Xiang J. Z. Anderson S. A. (2008). A spatial bias for the origins of interneuron subgroups within the medial ganglionic eminence. Dev. Biol. 314, 127–136. 10.1016/j.ydbio.2007.11.018 18155689 PMC2727678

[B228] Xu Q. Cobos I. De La Cruz E. Rubenstein J. L. Anderson S. A. (2004). Origins of cortical interneuron subtypes. J. Neurosci. 24, 2612–2622. 10.1523/JNEUROSCI.5667-03.2004 15028753 PMC6729522

[B229] Xu Q. Tam M. Anderson S. A. (2008). Fate mapping Nkx2.1-lineage cells in the mouse telencephalon. J. Comp. Neurol. 506, 16–29. 10.1002/cne.21529 17990269

[B230] Xu Q. Guo L. Moore H. Waclaw R. R. Campbell K. Anderson S. A. (2010). Sonic hedgehog signaling confers ventral telencephalic progenitors with distinct cortical interneuron fates. Neuron 65, 328–340. 10.1016/j.neuron.2010.01.004 20159447 PMC2868511

[B231] Yabut O. Renfro A. Niu S. Swann J. W. Marin O. D'Arcangelo G. (2007). Abnormal laminar position and dendrite development of interneurons in the reeler forebrain. Brain Res. 1140, 75–83. 10.1016/j.brainres.2005.09.070 16996039

[B232] Yau H. J. Wang H. F. Lai C. Liu F. C. (2003). Neural development of the neuregulin receptor ErbB4 in the cerebral cortex and the hippocampus: preferential expression by interneurons tangentially migrating from the ganglionic eminences. Cereb. Cortex 13, 252–264. 12571115 10.1093/cercor/13.3.252

[B233] Yoneda A. Couchman J. R. (2003). Regulation of cytoskeletal organization by syndecan transmembrane proteoglycans. Matrix Biol. 22, 25–33. 12714039 10.1016/s0945-053x(03)00010-6

[B234] Yozu M. Tabata H. Nakajima K. (2004). Birth-date dependent alignment of GABAergic neurons occurs in a different pattern from that of non-GABAergic neurons in the developing mouse visual cortex. Neurosci. Res. 49, 395–403. 10.1016/j.neures.2004.05.005 15236865

[B235] Yozu M. Tabata H. Nakajima K. (2005). The caudal migratory stream: a novel migratory stream of interneurons derived from the caudal ganglionic eminence in the developing mouse forebrain. J. Neurosci. 25, 7268–7277. 10.1523/JNEUROSCI.2072-05.2005 16079409 PMC6725225

[B236] Yun K. Fischman S. Johnson J. Hrabe de Angelis M. Weinmaster G. Rubenstein J. L. (2002). Modulation of the notch signaling by Mash1 and Dlx1/2 regulates sequential specification and differentiation of progenitor cell types in the subcortical telencephalon. Development 129, 5029–5040. 12397111 10.1242/dev.129.21.5029

[B237] Yun K. Garel S. Fischman S. Rubenstein J. L. (2003). Patterning of the lateral ganglionic eminence by the Gsh1 and Gsh2 homeobox genes regulates striatal and olfactory bulb histogenesis and the growth of axons through the basal ganglia. J. Comp. Neurol. 461, 151–165. 10.1002/cne.10685 12724834

[B238] Zhang X. F. Schaefer A. W. Burnette D. T. Schoonderwoert V. T. Forscher P. (2003). Rho-dependent contractile responses in the neuronal growth cone are independent of classical peripheral retrograde actin flow. Neuron 40, 931–944. 14659092 10.1016/s0896-6273(03)00754-2

[B239] Zhang W. Kang J. S. Cole F. Yi M. J. Krauss R. S. (2006). Cdo functions at multiple points in the Sonic Hedgehog pathway, and Cdo-deficient mice accurately model human holoprosencephaly. Dev. Cell 10, 657–665. 10.1016/j.devcel.2006.04.005 16647303

[B240] Zhao Y. Marin O. Hermesz E. Powell A. Flames N. Palkovits M.. (2003). The LIM-homeobox gene Lhx8 is required for the development of many cholinergic neurons in the mouse forebrain. Proc. Natl. Acad. Sci. U.S.A. 100, 9005–9010. 10.1073/pnas.1537759100 12855770 PMC166428

[B241] Zhao Y. Flandin P. Long J. E. Cuesta M. D. Westphal H. Rubenstein J. L. (2008). Distinct molecular pathways for development of telencephalic interneuron subtypes revealed through analysis of Lhx6 mutants. J. Comp. Neurol. 510, 79–99. 10.1002/cne.21772 18613121 PMC2547494

[B242] Zimmer G. Garcez P. Rudolph J. Niehage R. Weth F. Lent R.. (2008). Ephrin-A5 acts as a repulsive cue for migrating cortical interneurons. Eur. J. Neurosci. 28, 62–73. 10.1111/j.1460-9568.2008.06320.x 18662335

[B243] Zimmer G. Rudolph J. Landmann J. Gerstmann K. Steinecke A. Gampe C.. (2011). Bidirectional ephrinB3/EphA4 signaling mediates the segregation of medial ganglionic eminence- and preoptic area-derived interneurons in the deep and superficial migratory stream. J. Neurosci. 31, 18364–18380. 10.1523/JNEUROSCI.4690-11.2011 22171039 PMC6623906

